# Rapamycin, Acarbose and 17α-estradiol share common mechanisms regulating the MAPK pathways involved in intracellular signaling and inflammation

**DOI:** 10.1186/s12979-022-00264-1

**Published:** 2022-02-01

**Authors:** Lily Wink, Richard A. Miller, Gonzalo G. Garcia

**Affiliations:** 1 Department of Chemistry, University of Michigan College of Literature Science and The Arts, Ann Arbor, USA; 2 Department of Pathology, University of Michigan School of Medicine, Ann Arbor, USA; 3 University of Michigan Geriatrics Center, Room 3005 BSRB, Box 2200, 109 Zina Pitcher Place, Ann Arbor, MI 48109-2200 USA

**Keywords:** Aging, Inflammation, Diets, Rapamycin, Acarbose, 17-alpha-estradiol, Signal transduction, Acute phase proteins, Liver and kidneys

## Abstract

**Background:**

Rapamycin (Rapa), acarbose (ACA), and 17α-estradiol (17aE2, males only) have health benefits that increase lifespan of mice. Little is known about how these three agents alter the network of pathways downstream of insulin/IGF1 signals as well as inflammatory/stress responses.

**Results:**

ACA, Rapa, and 17aE2 (in males, but not in females) oppose age-related increases in the MEK1- ERK1/2-MNK1/2 cascade, and thus reduce phosphorylation of eIF4E, a key component of cap-dependent translation. In parallel, these treatments (in both sexes) reduce age-related increases in the MEK3-p38MAPK-MK2 pathway, to decrease levels of the acute phase response proteins involved in inflammation.

**Conclusion:**

Each of three drugs converges on the regulation of both the ERK1/2 signaling pathway and the p38-MAPK pathway. The changes induced by treatments in ERK1/2 signaling are seen in both sexes, but the 17aE2 effects are male-specific, consistent with the effects on lifespan. However, the inhibition of age-dependent p38MAPK pathways and acute phase responses is triggered in both sexes by all three drugs, suggesting new approaches to prevention or reversal of age-related inflammatory changes in a clinical setting independent of lifespan effects.

**Supplementary Information:**

The online version contains supplementary material available at 10.1186/s12979-022-00264-1.

## Background

Several dietary and pharmacological treatments extend mouse lifespan, including rapamycin (Rapa, [1]) and acarbose [ACA, [2]] in both sexes, and 17α-estradiol in males only [17aE2, [2, 3]]. These longevity experiments were done using the genetically heterogeneous UM-HET3 mouse stock, to avoid effects limited to single inbred backgrounds. However, the intracellular mechanisms regulated by these drugs are not well understood. Rapa inhibits the activity of the mammalian target of rapamycin (mTOR), leading at optimal doses to 20–25% lifespan extension in male and female mice [4–7] and prevents many forms of age-dependent pathology, including effects of aging on liver, heart, tendon, and kidney [8]. ACA is an inhibitor of α-glucosidase hydrolase enzymes and a-amylases, enzymes that digest carbohydrates in the small intestine. Thus, mice treated with ACA have a reduction in the pace of glucose absorption and in peak post-prandial glucose levels in blood [9, 10]. ACA extends median lifespan by 22% in males and 5% in females (significant at *p* = 0.01) and has been shown to have major beneficial effects in glucose homeostasis affecting multiple tissues. ACA exposure also reduces mTOR signaling in liver and kidney tissues [2, 8]. 17aE2 is a non-feminizing steroid that has a reduced affinity for the classical estrogen receptors [2]. 17aE2 has reproducible and robust effects, including an increase in male lifespan, and leads to beneficial effects in muscle and declines in mTOR signaling in males only [8, 11]. 17aE2 does not, however, lead to significant effects on female lifespan. The basis for sexual dimorphism in the effects of 17aE2 on lifespan extension is unknown [3]. Each of these agents is known to modulate the levels of hormones, such as IGF1, and insulin in blood as well as expression of inflammatory cytokines in plasma [12–18]. Rapa treatments starting at 20 months of age mice have beneficial effect on lifespan similar to those seen in mice exposed to the drug from 9 months of age [19, 20]. In addition, 17aE2 can improve some age-sensitive functions even when treatments are started at 16 months of age [21]. These results suggested that treatments starting in middle age can affect fundamental pathways involved in lifespan extension. mTOR forms two complexes, mTORC1 and mTORC2. Data from our lab have suggested that mTORC1 is diminished by each of these three drugs, whether treatment is started at 6 or at 22 months of age). All three drugs also induce declines in cap-dependent translation and enhanced cap-independent translation (CIT) via enhanced expression of the eukaryotic translation initiation factor 4E-binding protein 1 in liver and kidney tissues [8] (4EBP1). The increases in 4EBP1 have been suggested to have beneficial effects in mice [22, 23]. Our lab has also found that Rapa, ACA and 17aE2 treatments can enhance mTORC2 signaling in liver [11], but the implication of this mTORC2 effect for the aging process is not understood.

**Fig. 1:**
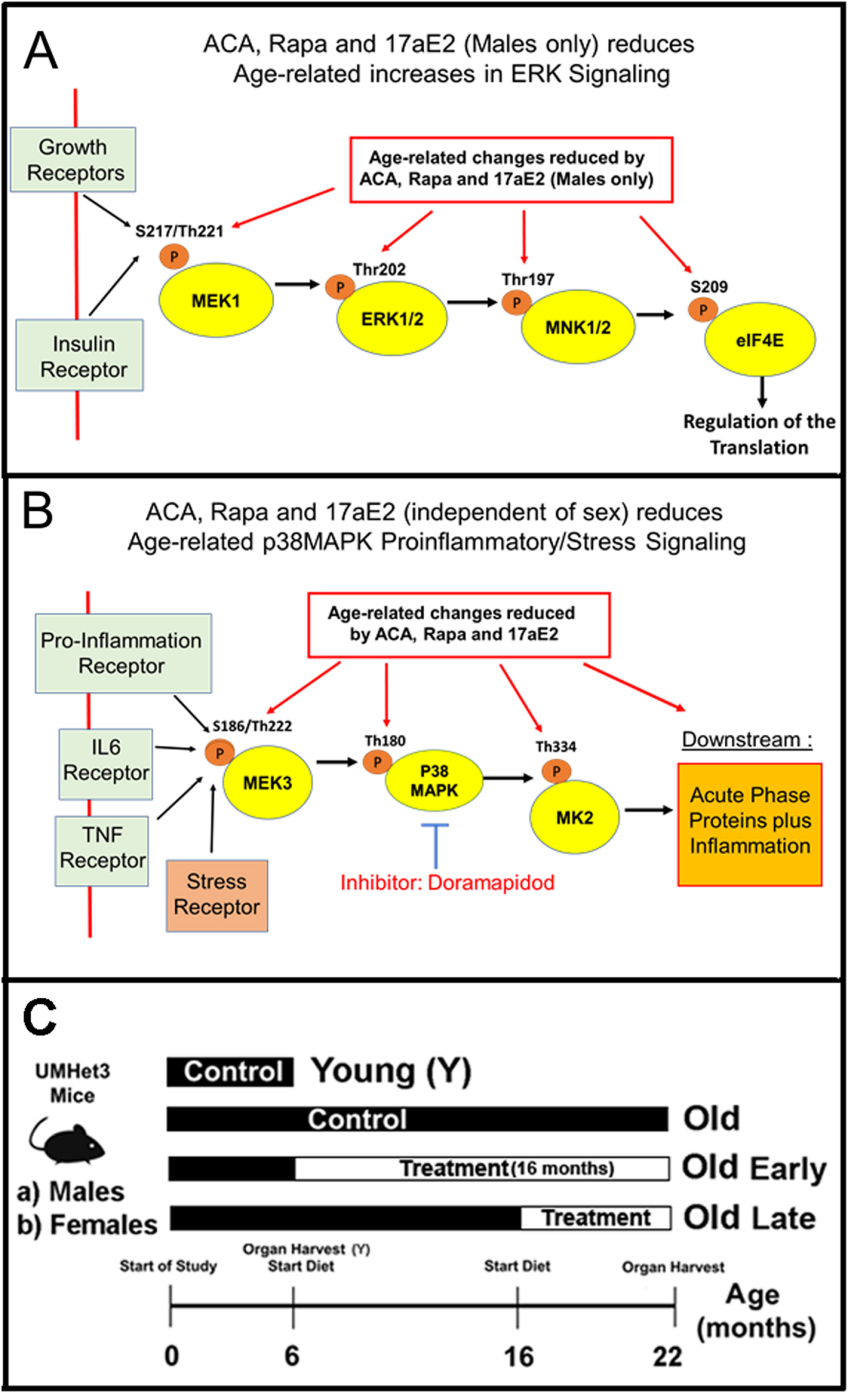
**A** Diagram of the MEK1/ERK signaling pathway study**. B** Diagram of the p38MAPK signaling pathway study. **C** Diagram of the experimental design for the treatments

The data showing similar effects of Rapa, ACA and 17aE2 on mTORC1 and its downstream targets - including stimulation of CIT- [8], suggested other common pathways, such as pathways mediated by AMPK [24] or MAPK pathways [25], might be affected by these agents. This paper focuses on two parallel sets of kinase pathways generally known as the MAPK signaling cascades, one of which is initiated by MEK1 phosphorylation of ERK1/2, and the other of which is initiated by MEK3 phosphorylation of p38-MAPK. The ERK1/2 signaling pathway can respond to extracellular growth factors, including insulin and IGF1 [26]. In liver and kidneys, growth factors activate the ERK1/2 pathway by phosphorylation of the dual specificity mitogen-activated protein kinase-kinase 1 (MEK1) on Serine 217/Threonine 221 (pMEK1), via activation of the RAF signaling and possibly by other non-canonical signals [26]. In turn, activation of MEK1 leads to activation of the mitogen-activated protein kinase 3 (ERK1) and mitogen-activated protein kinase 1 (ERK2) by phosphorylation of threonine 202 (pERK1) and threonine 185 (pERK2) respectively. Activated ERK1/2 then leads to phosphorylation of the MAP kinase-interacting serine/threonine-protein kinases 1 and 2 (MNK1 and 2) at their threonine 197/202 site (pMNK). In mice, MNK1 is a single isoform while MNK2 includes two isoforms, MNK2a and MNK2b; little is known about possible differences in function and expression of these two MNK2 isoforms in mouse tissues [25] One key function of MNK, among many [25], is the phosphorylation of the elongation and initiation factor 4E (eIF4E) at serine 209 (peIF4E). This phosphorylation site is known to control the translation of specific subsets of mRNA. Interestingly, the specific mechanism is not understood, and it is possible that peIF4E may regulate, indirectly, the cap independent translation or CIT, [27]. These relationships are depicted in Fig. 1A. In particular, our previous data have shown that ACA, Rapa and 17aE2 treatments can enhance CIT, via reduction in mTOR signaling [8], suggesting that these drugs might also affect the MEK1 kinase pathway, including MNKs, that leads to eIF4E phosphorylation.

The parallel MEK3 kinase cascade responds to extracellular inflammatory factors, such as TNF, IL6 and cytokines, as well as to intracellular stress signaling stimuli [25], such as DNA damage [28]. Activation of this pathway is therefore very complex, depending on the tissue and specific receptor(s) triggered. However, many of these stressors and inflammatory factors converge on the activation of dual specificity mitogen-activated protein kinase kinase 3 (MEK3) by phosphorylation on serine 186 [pMEK3, [25]]. The activation of MEK3 leads to a cascade of events that includes the activation of members of the p38-MAPK kinase family by phosphorylation on threonine 180 [pp38, [25]]. Examples include MAPK11 (p38α) and MAPK14 (p38β). In turn, phosphorylated p38 kinases can phosphorylate and activate a myriad of substrates, including the MAP kinase-activated protein kinase 2 (MK2) at threonine 334 [pMK2, [29]]. MK2 activation has been implicated in many inflammatory and stress processes [29] as well as in the phenomenon of cell senescence [30, 31]. In this context, there are data suggesting that rapamycin can reduce the production of harmful secreted products from senescent cells by reducing the activity of MK2 [30, 31]. In vitro, MK2 is present in two isoforms, MK2-short and MK2-long. The MK2-long isoform is generated by in frame alternative 5′ UTR translation from the same mRNA transcript that encodes the MK2-short isoform [32]. It is unclear what processes govern the relative levels of each isoform [32], but both isoforms can be phosphorylated by p38. Current evidence suggests that the MK2-short form, but not the MK2-long form, may regulates downstream proinflammatory responses [32].

Downstream targets of the p38/MK2 cascade are extensive [33] and include elements of the DNA repair machinery [34], cellular senescence [35] and the mRNA binding proteins regulating mRNA level proinflammatory cytokines [36]. Inflammatory mediators, such as IL-6, are known to regulate the expression of the acute phase response proteins or APPs [37]. In human and mouse, proinflammatory cytokines and related stress events are transient and difficult to quantify at the protein level, but APPs are abundant and stable proteins that have protective effects. High levels of APPs are considered markers of a proinflammatory or stress phenotype, in mice and humans, that if unchecked would lead to long-term tissue damage [38, 39]. These APPs include serum amyloid P-component (SAP), Alpha-1-acid glycoprotein 1 (ORM1), the serpin family of protease inhibitors [Alpha-1-antitrypsin 1–4 protease inhibitor 1–4 (Serpin A1d) and Alpha-1-antitrypsin 1–5 (Serpin A1e)], as well as Heme oxygenase 1 and 2 (HMOX1/2) and several caspases, such as caspase 6 (Cas6) and caspase 3 (Cas3). These relationships are depicted in Fig. 1 B. To see if the beneficial effects of ACA, Rapa, and 17aE2 might involve a reduction in tissue damage and inflammatory injury, we initiated a study of the effects of age and drug effects on the p38MAPK signals that lead to changes in the levels of APPs.

Recently, we have found that ACA and Rapa, in both sexes, and 17aE2 (in males only) can reduce mTORC1 signaling, suggesting that pathways upstream of mTOR may be affected by these drugs, potentially including mediators of IGF-1 and insulin signaling [8] or other extracellular factors that often act via one of the MAPK (MEK1 or MEK3) cascades. In this report we document the effects of these three agents on two such kinase cascades. We find that the MEK3 pathway, leading to APP production. is down-regulated by all three agents in both sexes, but that the MEK1 pathway, leading to eIF4E phosphorylation, while down-regulated by Rapa and ACA in both sexes, is inhibited by 17aE2 in male mice but not in females. Thus, the pattern of sexual dimorphism in lifespan extension parallels the outcome of MEK1 signals, but not MEK3 signals. Anti-inflammatory effects of interference by these drugs on inflammatory signals might potentially generate health benefits in both sexes.

## Results

### Age-related increases in the MEK1-ERK-MNK-eIF4E signaling pathway are downregulated by ACA, Rapa and 17aE2

To evaluate effects of age and anti-aging drugs on the MEK1 kinase cascade, we collected lysates from livers and kidneys of untreated young mice (6 months of age, Y), untreated old mice (22 month of age, O) and mice treated with either ACA, Rapa or 17aE2 from 6 to 22 months of age (O, Early) or from 18 to 22 months of age (O, Late). Supplemental Fig. 1 C shows a visualization of the experimental design. Two-factor ANOVA was used, for each endpoint, to evaluate possible sex-specific effects of each drug No significant interaction terms were found for mice treated with Rapa or ACA, but effects of 17aE2 were significantly different by sex for some endpoints (see Supplemental Table 1). For this reason, data for Rapa and ACA were evaluated without respect to sex, but 17aE2 results were evaluated separately for each sex, as in our previous study of the effects of these three drugs on mTORC1 action and CIT protein levels [8].

**Fig. 2:**
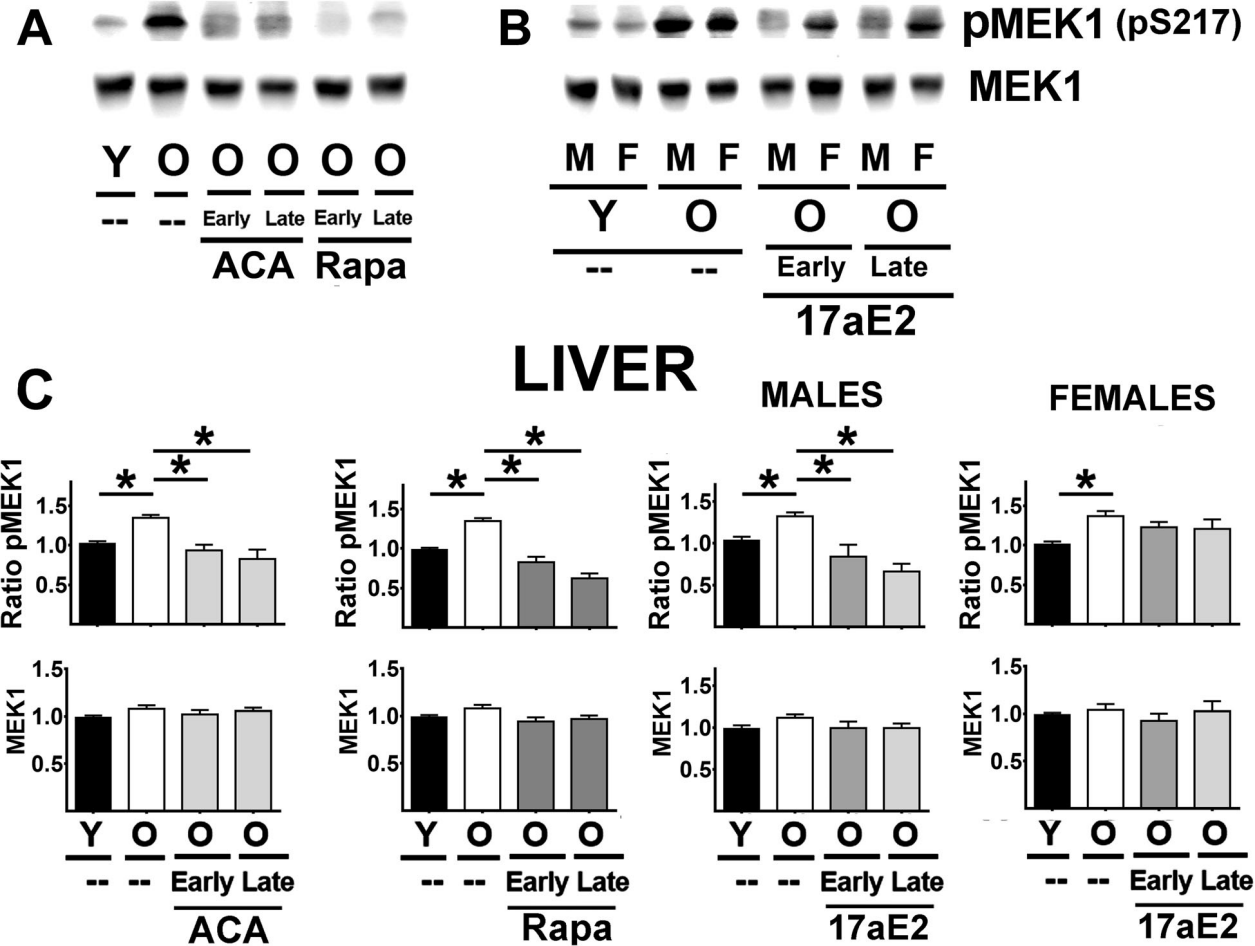
Effect of ACA, Rapa and 17aE2 treatments on liver MEK1 signaling in liver. **A** Representative western blots for phospho-MEK1 at Ser217 (pMEK1) and total levels of MEK1 protein in liver samples from young untreated (Y), old untreated (O), and old mice treated with ACA or Rapa (O, early and late treatments). **B** Representative western blots for pMEK1 and MEK1 effects of 17aE2 (early and late) treatments separated by sex (males = M, females = F). **C** Bar graphs represent the mean ± SEM of pMEK1 ratios and MEK1 protein levels in liver samples obtained from 16 young and 16 old mice plus, at least, 8 mice for each of the ACA and Rapa treatments groups. Data for 17aE2 were obtained from 8 young males and 8 young females, 8 old males and 8 old female mice, while treatments represent a minimum of 4 mice for each treatment and sex group. All values have been normalized to young control females as described in the Methods section. The (*) indicates statistical significance (*p* < 0.05) in a t-test between the indicated pair of groups

**Fig. 3:**
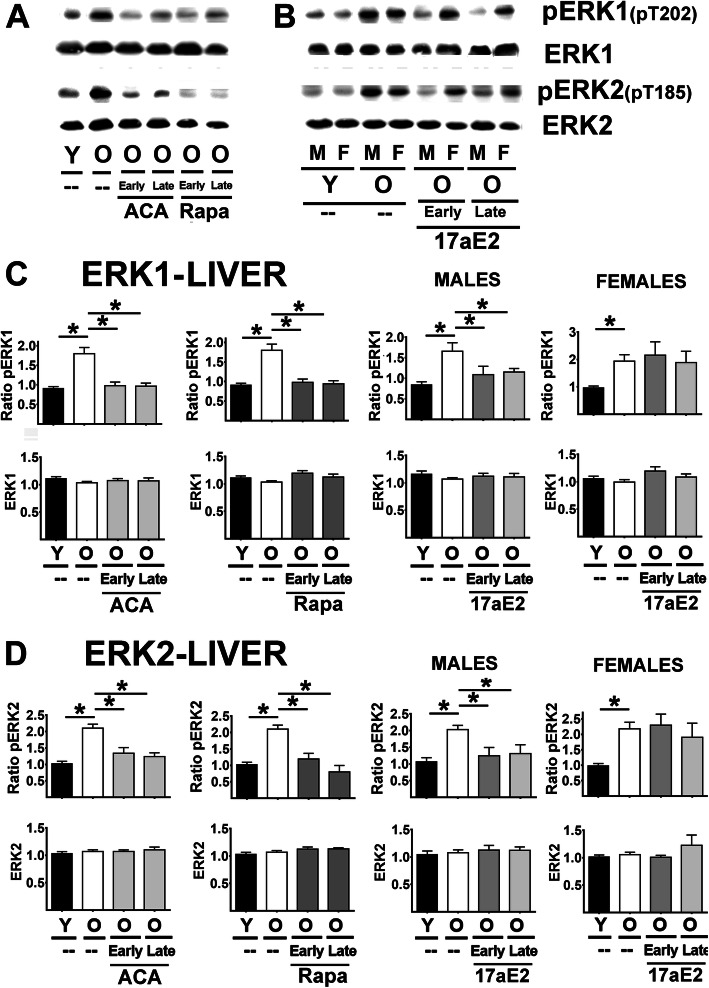
Effect of ACA, Rapa and 17aE2 treatments on liver ERK1 and ERK2 signaling in liver. **A** Representative western blots for phospho-ERK1 at Threonine 202 (pERK1), phosphor-ERK2 at Threonine 185 (pERK2) and total levels of ERK and ERK2 protein in liver samples from young untreated (Y), old untreated (O), old treated with ACA or Rapa (O, early and late treatments). **B** Same as A with 17aE2 treatment separated by sex (males = M, females = F). **C**) Bar graphs represent the mean ± SEM of pERK1 ratios and ERK1 protein levels in liver samples obtained from 16 young and 16 old mice plus, at least, 8 mice for each of the ACA and Rapa treatments groups. Data for 17aE2 was obtained from 8 young males and 8 young females, 8 old males and 8 old female mice, while treatments represent a minimum of 4 mice for each treatment and sex group. All values have been normalized to young control females as described in the Methods section. The (*) indicates statistical significance (*p* < 0.05) in a t-test between the indicated pair of groups. **D** Bar graphs represent the mean ± SEM for pERK2 ratios and ERK2 protein levels in kidney samples as described above

Aging led to an increase in the ratio of pMEK1 to total MEK1 but did not alter MEK1 protein levels in liver (Fig. 2A-C) or kidney (Supplemental Fig. 1A). The increased proportion of phosphorylated MEK1 was opposed by both Rapa and ACA, regardless of the age at which treatment was started (Fig. 2 C and Sup. Figure 1). 17aE2 showed the same effects in males but had no significant effects in females for either liver or kidney samples (see Fig. 2 and Sup. Figure 1 Females right panels). A parallel analysis of ERK1, a target of MEK1, produced very similar results (liver in Fig. 3 and kidney in Sup. Figure 1 B): an age-related increase in the ratio of pERK1 (T202) to total ERK1 protein, without any age effect on total ERK1 protein levels. The age effects were opposed by Rapa and ACA in both sexes and by 17aE2 in males only, with parallel results in liver and kidney. There was one minor exception to this pattern: the reduction in the ratio of pERK1 for late 17aE2 treatments in kidney did not reach statistical significance. ERK2 followed the same pattern as ERK1, with age-related increases in ratio of pERK2 (liver Fig. 3 D, kidney Sup. Figure 1 C), significantly diminished by ACA and Rapa treatments (in both early and late treatments); these changes reflect a significant reduction in the ratio of pERK2 with no changes in ERK2 proteins levels. 17aE2 led to similar effects in males but had no effects in females (Fig. 3 D and Sup. Figure 1 C, Females right bar graphs).

**Fig. 4:**
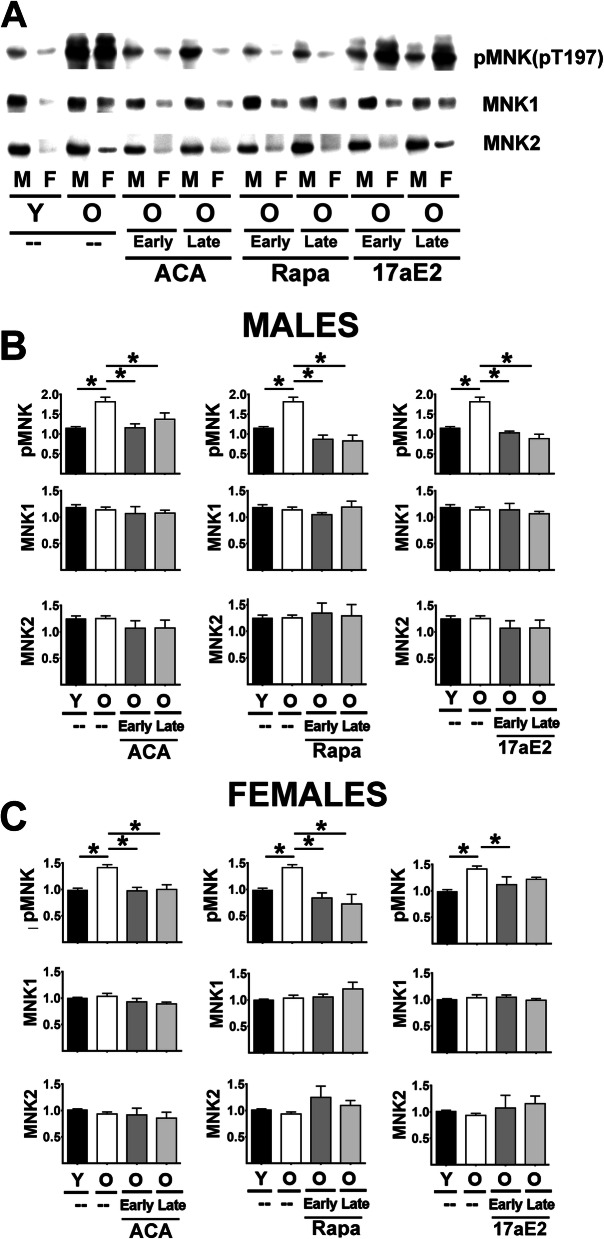
Effect of ACA, Rapa and 17aE2 treatments on liver MNK signaling in liver. **A** Representative western blots for phospho-MNK at Threonine 197 (pMNK) and total levels of MNK1 and MNK2 protein in liver samples from young untreated (Y), old untreated (O), old treated with ACA or Rapa (O, early and late) and 17aE2 (early and late) treatments separated by sex (males = M, females = F). **B** Bar graphs represent the mean ± SEM of pMNK and MNK1 and MNK2 protein levels in liver of 8 young and 8 old male mice plus, at least, 4 mice for each of the ACA and Rapa and 17aE2 male treated groups. All values have been normalized to young control females as described in Methods section. **C** Bar graphs represent the mean ± SEM of pMNK and MNK1 and MNK2 protein levels in liver of 8 young and 8 old female mice plus, at least, 4 mice for each of the ACA and Rapa and 17aE2 female treated groups. The (*) indicates statistical significance (*p* < 0.05) in a t-test between the indicated pair of groups

The key downstream target of ERK1/2 is the MNK family of kinases. Figure 4 A shows a typical western blot of MNK1 and MNK2 protein in liver tissues. MNK1 appears as a single band and MNK2 also shows a single band at a molecular weight corresponding to that expected for the MNK2a splice isoform [40]. The MNK2b alternative splice isoform may be at low levels in the liver or not detected by the antibody used. Tests for MNK2a and MNK2b mRNA, using previously reported isoform-specific probes for mouse [40], shows MNK2b CT values above 34 suggesting that this isoform may not be significantly expressed in liver or kidneys (Supplemental Fig. 2A, lower panels). Phosphorylation of MNK at Threonine 197/202 (pMNK) in liver samples produces a single band (Fig. 4A). Phosphorylation sites of MNK1 and MNK2 predicted by its sequence should be indistinguishable by western blots, making it impossible to calculate ratios of phospho-protein to total protein for either isoform, and therefore statistical analysis was done on pMNK independently of the levels of MNK1 or MNK2 protein.

Two-way Anova revealed, unexpectedly, that MNK1 and MNK2 protein levels are higher in males than in females for both liver and kidney samples (Supplemental Table 1). This sexual dimorphism for MNK1 and MNK2 was confirmed by mRNA levels (Supplemental Fig. 2A/B), suggesting that it results, at least in part, from transcriptional regulation. We therefore analyzed the effects of age and treatments on MNK and its phosphoproteins separately for each sex. Aging led to an increase in pMNK in both sexes, without a change in levels of MNK1 or MNK2 (See Fig. 4B in males and Fig. 4C in females). Whether started early or late, ACA, Rapa and 17aE2 show a significant reduction in pMNK in male mice, with no effects on MNK1 or MNK2 protein levels. Females resembled males in their responses to Rapa and ACA, but the pMNK response to 17aE2 was significant only when 17aE2 was started early in adult life. Analysis of kidney samples (Supplemental Fig. 3A for males and Fig. 3 B for females) produced essentially the same pattern seen in liver, with age-related increases in pMNK in both sexes, and reduction of pMNK by ACA and Rapa in both sexes. 17aE2 effects in kidney were seen only in males.

**Fig. 5:**
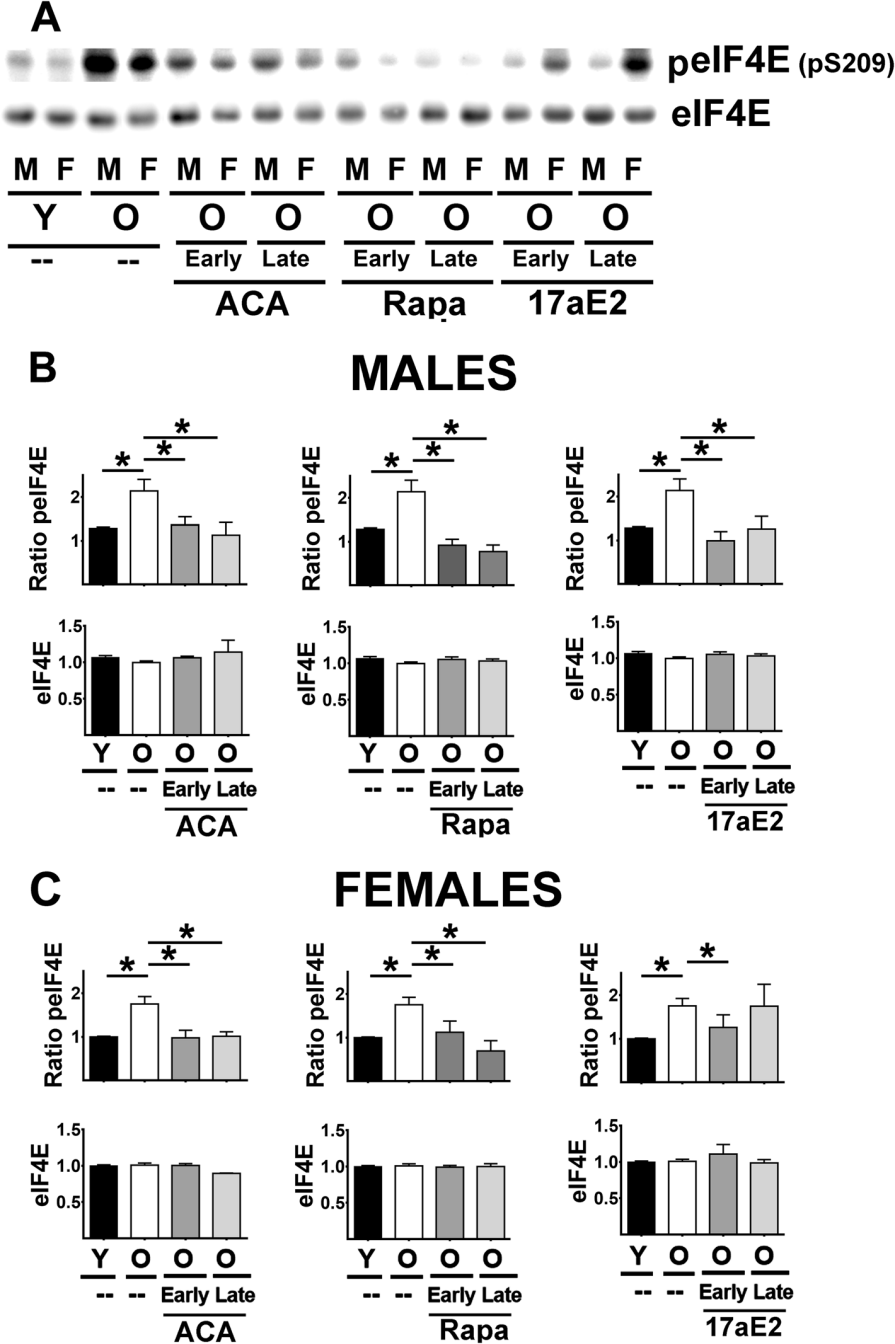
Effect of ACA, Rapa and 17aE2 treatments on liver eIF4E signaling in liver. **A** Representative western blots for phospho-eIF4E at Serine 209 (peIF4E) and total levels of eIF4E protein in liver samples from young untreated (Y), old untreated (O), old treated with ACA or Rapa (O, early and late) and 17aE2 (early and late) treatments separated by sex (males = M, females = F). **B** Bar graphs represent the mean ± SEM of peIF4E ratio and eIF4E protein levels in liver of 8 young and 8 old male mice plus, at least, 4 mice for each of the ACA and Rapa and 17aE2 male treated groups. All values have been normalized to young control females as described in the Methods section. **C** Bar graphs represent the mean ± SEM of peIF4E ratio and eIF4E protein levels in liver of 8 young and 8 old female mice plus, at least, 4 mice for each of the ACA and Rapa and 17aE2 female treated groups. The (*) indicates statistical significance (*p* < 0.05) in a t-test between the indicated pair of groups

MNK, once activated, phosphorylates eIF4E at Ser 209 (peIF4E), a critical step in cap-dependent translation. Two-way Anova showed that males have higher levels of peIF4E than females for both liver and kidney samples (Supplemental Table 1). Therefore, we performed an analysis by age and treatments in each sex separately. The results paralleled those seen for pMNK: age-associated increases in peIF4E in both sexes, without any change in total eIF4E, blocked by Rapa and ACA in both sexes and by 17aE2 in males, in both liver (Fig. 5B) and kidney (Supplemental Fig. 3C). Effects of 17aE2 in females were less consistent than in males (Fig. 5C and Sup. Figure 3C), but similar to those noted for pMNK: this agent lowered peIF4E only in liver, and only if started early in life. None of the treatments modified eIF4E protein levels.

### Age-related increases in p38-MAPK signaling are downregulated by ACA, Rapa and 17aE2 in both sexes

The second chain of kinase-dependent activation events shown in Fig. 1 B is initiated by inflammatory receptors, such as IL-6R, TNFR and stress stimuli, that lead to the activation of MEK3, p38-MAPK, and MK2 signaling, resulting in the synthesis and secretion of acute phase proteins and other indices of inflammation [26]. Supplemental Table 2 displays statistical analysis, using two-way ANOVA, for each element in this p38-MAPK signaling pathway, to determine effects of age, sex, drug effects (Rapa, ACA, 17aE2), [age x sex] and [drug x sex] interactions, and drug effects (ACA, Rapa and 17aE2) on levels of total protein and phosphorylation status for MEK3, p38-MAPK and MK2. As we had noted for the proteins in the MEK1 cascade (shown in Supplemental Table 1), we saw no significant sex interaction terms for Rapa or ACA, and therefore males and females were combined for analyses of these two drugs. In contrast with the elements of the MEK1 cascade, 17aE2 treatments showed no sex-specific effects, i.e. no significant sex-interaction terms in the Anova (Supplemental Table 2). Because 17aE2 influences lifespan in male mice only [2], we decided to present the 17aE2 data separately for each sex.

**Fig. 6:**
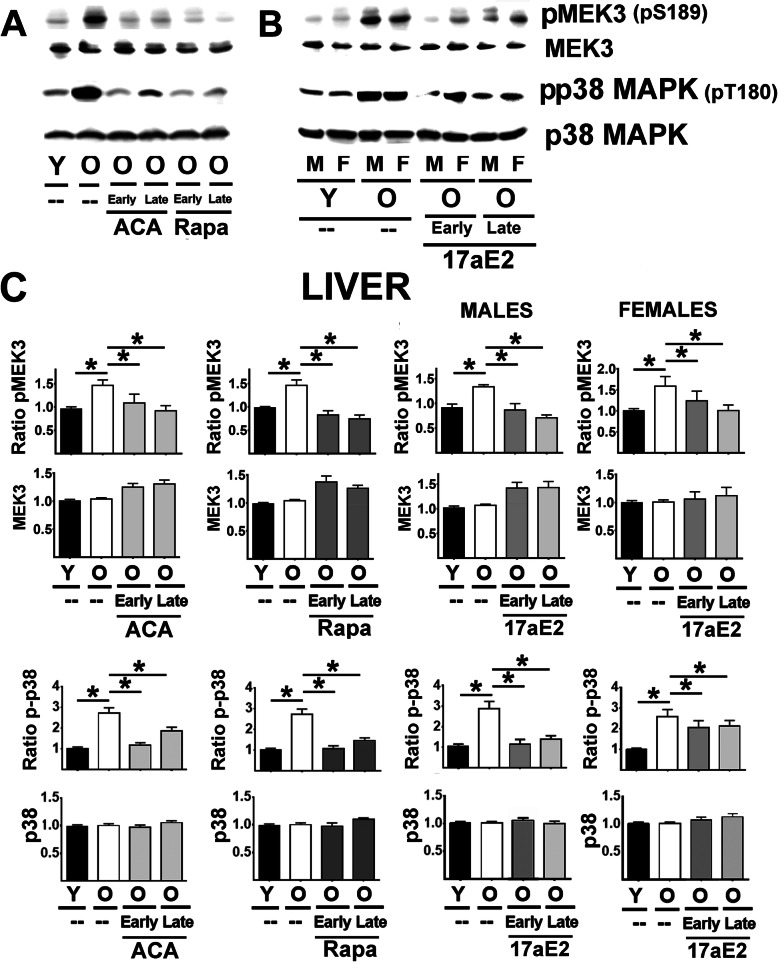
Effect of ACA, Rapa and 17aE2 treatments on liver MEK3 and p38MAPK signaling in liver. **A** Representative western blots for phospho-MEK3 at Ser189 (pMEK3) and total levels of MEK3 protein as well as phosphor-p38MAPK at Threonine 180 (p-p38) and p38 protein levels in liver samples from young untreated (Y), old untreated (O), old treated with ACA or Rapa (O, early and late treatments). **B** Representative western blots of 17aE2 (early and late) effects on pMEK3, MEK3, p-p38 and p38 protein levels separated by sex (males = M, females = F). **C** Bar graphs represent the mean ± SEM of pMEK3 ratios and MEK3 protein levels in liver samples from 16 young and 16 old mice plus, at least, 8 mice for each of the ACA and Rapa treatments groups. The 17aE2 was from 8 young males and 8 young females, 8 old males and 8 old female mice, while treatments represent a minimum of 4 mice for each treatment and sex group. All values have been normalized to young control females as described in the Methods section. The (*) indicates statistical significance (*p* < 0.05) in a t-test between the indicated pair of groups. **D** Bar graphs represent the mean ± SEM for p-p38 ratios and p38 protein levels as described above

Figure 6 (top panels A and B) show a typical western blot of MEK3 protein levels and its phosphorylated form (pMEK3 at S217) in liver samples. Analysis of liver (Fig. 6C, top panels) and kidney samples (Supplemental Fig. 4A) showed a significant age-related increase in the ratio of pMEK3/MEK3 but no significant changes in MEK3 protein levels. All three drugs, Rapa, ACA, and 17aE2 led to a significant reduction in the ratio of pMEK3/MEK3 with no changes in MEK3 protein levels and did so in both sexes and regardless of the age of first exposure to the drug. These results contrast with the results for the MEK1-triggered cascade (Figs. 1, 2, 3, 4, and 5), where the effects of 17aE2 were strong only in males.

**Fig. 7:**
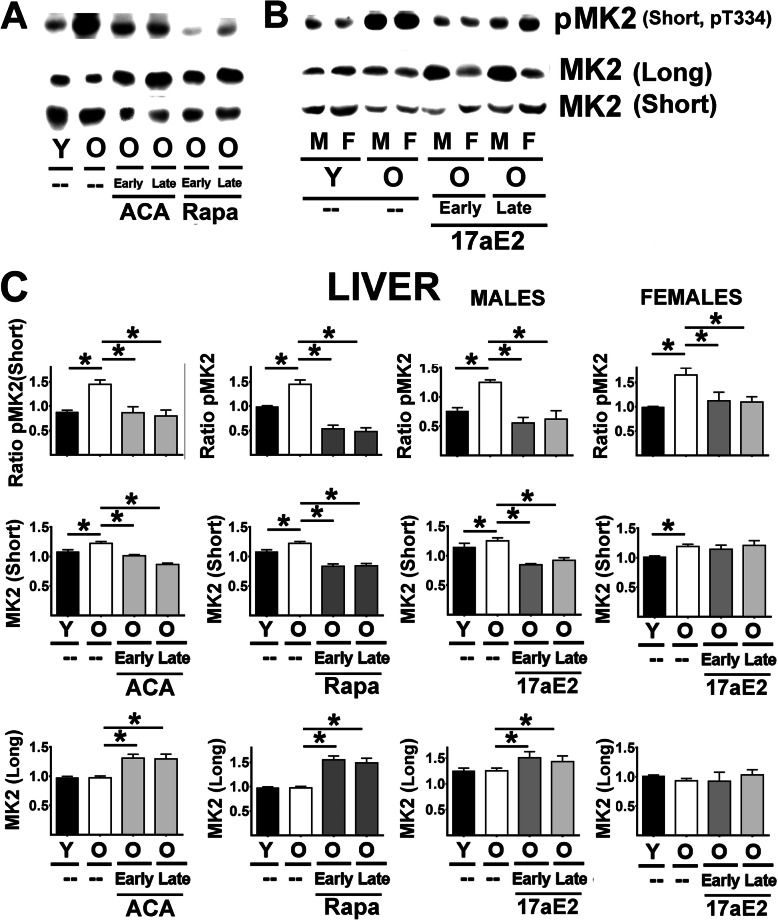
Effect of ACA, Rapa and 17aE2 treatments on MK2 signaling in liver. **A** Representative western blots for phospho-MK2 at Threonine 334 (pMK2), total levels of MK2 (Short) and MK2 (Long) protein isoforms in liver samples from young untreated (Y), old untreated (O), old treated with ACA or Rapa (O, early and late treatments). **B** Representative western blots of 17aE2 (early and late) effects in pMK2 and MK2 isoforms separated by sex (males = M, females = F). **C** Bar graphs represent the mean ± SEM of pMK2 ratios and MK2 (Long and Short) protein levels in liver (top panels) and kidney samples (bottom panels) obtained from 16 young and 16 old mice plus, at least, 8 mice for each of the ACA and Rapa treatments groups. 17aE2 was from 8 young males and 8 young females, 8 old males and 8 old female mice, while treatments represent a minimum of 4 mice for each treatment and sex group. All values have been normalized to young control females as described in Methods section. The (*) indicates statistical significance (*p* < 0.05) in a t-test between the indicated pair of groups

p38-MAPK (p38) is the key downstream target of MEK3. Figure 6A (lower panels) includes images from a typical western blot of p38 protein and its phosphorylated form (pp38) in liver samples. The results for analysis of liver pp38 (Fig. 6C, lower panels) were identical to those for MEK3: age associated increases in pp38 in both liver and kidney (Sup Figure. 4 B), inhibited by Rapa, ACA, and 17aE2, with equal effects of 17aE2 in both sexes, and without significant changes in total p38 protein. Activation of p38-MAPK leads to activation of MK2 by phosphorylation at threonine 334. Mouse liver and kidney express two MK2 isoforms, referred to as MK2 (short) and MK2 (long), as seen on the western blot in Fig. 7 (panels A and B). Levels of pMK2 (short) could be identified using a monoclonal antibody that recognizes phosphorylation in the Thr-334 residue of MK2. Unambiguous quantification of phosphorylated MK2 (long), however, was not possible, because we noted multiple bands in the region of the gel corresponding to the pMK2 (long) isoform. We therefore used quantified pMK2 (short) as an index of MK2 activation by p38-MAPK. The results for the ratio of pMK2 (short) to total MK2 (short), show in Fig. 7 C top panels, were very similar for those just presented for MEK3 and p38-MAPK, i.e. an age-related increase that is inhibited by ACA, Rapa, and 17aE2 in both sexes. The total amount of MK2 (short) increased slightly (Fig. 7 C, middle panel), but significantly, with age in both tissues and in both sexes; thus, the age-associated increase in the ratio of pMK2 (short) to total MK2 (short) did not reflect a decline in total MK2 (short) protein. The age-related increase in the amount of MK2 (short) was inhibited by Rapa and ACA in both sexes, and by 17aE2 in males only.

In contrast, the total amount of MK2 (long) protein did not change with age. Surprisingly, however, MK2 (long) did increase significantly in mice treated with ACA, Rapa, or (in males) 17aE2 (Fig. 7C). Similar results were found in samples of kidney tissues (Supplemental Fig. 4C), with downregulation of pMK2 (short) by ACA, Rapa and 17eE2 treatments (independent of sex) and changes in the expression of MK2 (short and long isoforms) as described in liver.

### ACA, Rapa and 17aE2 (in both sexes) lowers the expression of acute phase proteins (APPs)

**Fig. 8:**
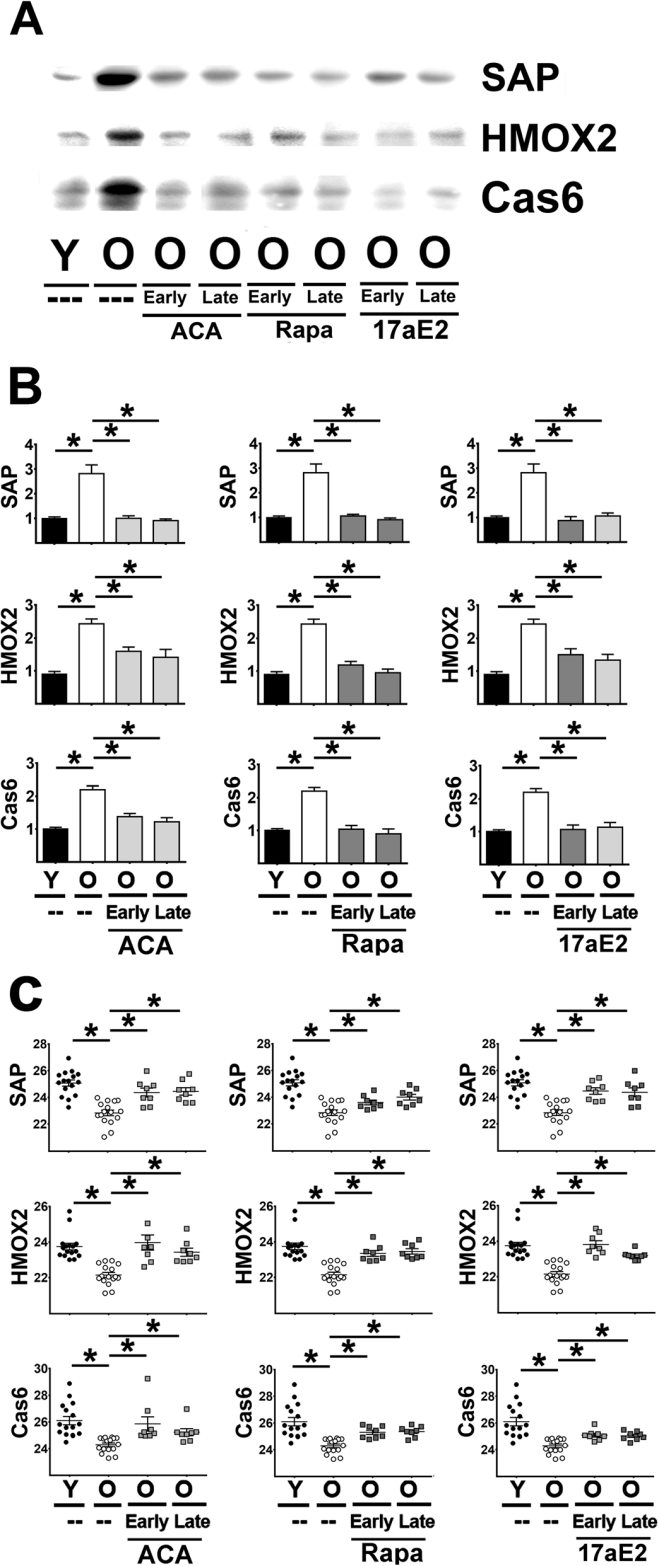
Effect of ACA, Rapa and 17aE2 treatments on the levels of Acute Phase Proteins in liver. **A** Representative western blots for SAP, HMOX2 and Cas6 in liver samples from young untreated (Y), old untreated (O), and old treated with ACA, Rapa and 17aE2 (O, early and late treatments). **B** Bar graphs represent the mean ± SEM of SAP, HMOX2 and Cas6 protein in liver samples obtained from 16 young and 16 old mice plus, at least, 8 mice for each of the ACA, Rapa and 17aE2 treatments groups. Values been normalized to young control females for each protein, as described in Methods section. The (*) indicates statistical significance (*p* < 0.05) in a t-test between the indicated pair of groups. **C** The scatter plot shows a comparison of CT values and mean ± SEM (horizontal bar) of each mRNA between the groups from 16 young and 16 old mice plus a minimum of 8 mice treated with ACA, Rapa and 17aE2 (earl or late) per group. Each symbol represents a different mouse. The (*) indicates statistical significance (*p* < 0.05) in a t-test between the indicated pair of groups

Declines in activation of pp38 and pMK2 are expected to produce effects on a wide range of downstream protein targets of this kinase cascade. Because the expression of many proinflammatory cytokines were likely to be too low and transient for reliable detection by western blots we measured instead levels of the APPs in our samples that should give a more reliable index of inflammation status. From the 40 proteins belonging to the APP pathway [33, 34], we measured three representative APPs (SAP, HOMX2 and Cas6) by immunoblotting (Fig. 8A/B) and using RT-PCR to measure the corresponding mRNA levels (Fig. 8C). Supplemental Table 3 shows the results of the two-way Anova for these endpoints. Aging led to an increase of all three of these APPs in liver (both protein in Fig. 8 B and mRNA levels in Fig. 8C), which was largely or completely inhibited by all three drugs in both sexes for both protein and mRNA (Fig. 8B/C). Kidney samples showed the same pattern (Supplemental 5 A for protein and B for mRNA). The inhibition of the age-related increases in the transcript levels for each of the three APPs by Rapa, ACA, and 17aE2, in both tissues and in both sexes, closely matches the effects of these three drugs on the p38/MK2 pathway, although it is possible that drug effects on other intermediary pathways, such as those involving NFkB [36], could also play a role.

### p38-MAPK regulates expression of APPs

**Fig. 9:**
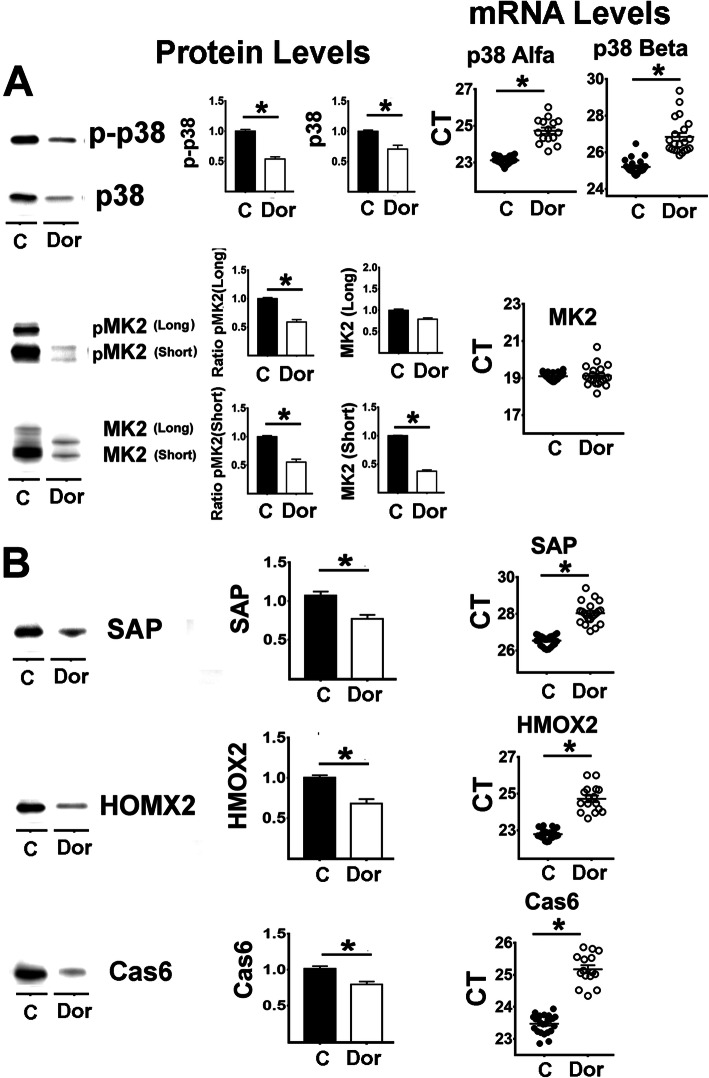
Effect of a p38MAPK inhibitor on the expression of Acute Phase Proteins in AML-12 cells. **A** Right panel shows effects of 48 h treatment with Doramapimod (Dor, p38MAPK inhibitor) on pMK2, MK2 protein levels, p-p38 levels and p38 protein levels. Middle panel shows the mean ± SEM for pMK2 and MK2 (Short and Long isoforms), p-p38 and p38 from 6 independent experiments. Right panels show the effects on mRNA levels for these proteins. **B** Effects of Dor on SAP, HMOX2 and Cas6 from the samples described above. The (*) indicates statistical significance (*p* < 0.05) in a t-test between the indicated pair of groups

To test the hypothesis that p38-MAPK can regulate levels of APPs we treated AML-12 cells (a well-documented model of hepatocytes [41]) with Doramapimod (“Dor”), a specific inhibitor of p38-MAPK [42] for 48 h, and then evaluated levels and phosphorylation status of p38-MAPK, long and short isoforms of MK2, APP, and their corresponding mRNAs. As shown in Fig. 9, Dor-treatments can diminish phosphorylation of p38-MAPK (Fig. 9A). The total amount of p38 protein also declined significantly, consistent with previous work [43, 44] suggesting that p38 and MK2 form a complex that stabilizes their levels, activity, and phosphorylation status. There is also evidence for transcriptional control of the p38/MK2 pathway by p38 inhibition. We noted lower levels of mRNA for both p38α and p38β isoforms (Fig. 9A, mRNA CT panel) in Dor-treated cells. Diminished p38 activity is expected to result in lower phosphorylation of MK2. In cultured AML-12 cells it is possible to visualize and quantitate both the long and the short forms of MK2 as well as their phosphorylated isoforms. As shown in Fig. 9A, pMK2 (long) and pMK2 (short) are both lower in Dor-treated cells. There is also a reduction in the level of MK2 (short) protein. The level of MK2 (long) protein is not changed, but the band migrates more rapidly than in control cells, consistent with a decline in its phosphorylation. qRT-PCR data show no effect of Dor-treatment on mRNA for MK2, suggesting that the changes in MK2 (short) protein may reflect post-transcriptional pathways.

Lastly, we evaluated levels of three APPs, i.e. SAP, HOMX2, and Cas6, in Dor-treated cells (Fig. 9B), All three proteins were significantly decreased, consistent with lowered p38 and MK2 activity. qRT-PCR data, also included in Fig. 9 right panels, showed a corresponding decline in mRNA for each of the three APPs, suggesting that the p38/MK2 pathway regulates APPs.

## Discussion

Our key finding is that the effects of three drugs that extend mouse lifespan fall into two subsets. Those which depend on the p38-MAPK cascade and lead to APP production are increased with age and blocked by all three drugs in both males and females. In contrast, those that depend on the ERK1/2 kinase cascade and lead to phosphorylation of eIF4E also increase with age and are blocked by Rapa and Aca in both sexes, but by 17aE2 only in male mice. Liver and kidney both show the same pattern of changes in both kinase pathways. The sexual dimorphism in the ERK1/2 pathway parallels the effects of these drugs on lifespan [2] and on several age-dependent aspects of health [11], suggesting that the blunting of age-dependent changes in the ERK1/2 pathway may itself contribute to the beneficial effects of all three drugs on health and survival. Lifespan responses to ACA repeatedly show greater benefit in male mice than in female mice, but effects of ACA on female survival have been significant and reproducible across replicate studies in independent cohorts [2]. Furthermore, the lack of sexual dimorphism in the p38-MAPK pathway suggests that pharmacological interventions that target this cascade could potentially blunt inflammatory effects of aging in both sexes.

Our previous work on kinase pathways focused on mutant mice in which dramatic lifespan extension was caused by down-regulation of GH and/or IGF-1 signals, such as the Snell Dwarf and GHR−/− deletion mice. These mice show improvement in many age-sensitive aspects of health, including glucose homeostasis, lipid metabolism and downregulation of proinflammatory responses [45, 46]. Snell and GHR−/− mice show downregulation of mTORC1 [47], ERK [48] and p38MAPK signaling [49, 50]. The declines in mTORC1 have been associated with lifespan increases and health benefits [51] and regulate translation of selective mRNAs by increases in cap-independent translation (CIT, [8, 52]. We speculated that these health benefits and changes in translation control might reflect alterations in one or more of the MAP kinase pathways. The canonical ERK signaling pathway regulates cell division [53], apoptosis [54, 55] , mitochondrial function [56], and many aspects of metabolism [57]. In contrast, the p38MAPK responds to many forms of stress, including DNA damage [58] and inflammatory signals [59], including IL-6 and TNF. Models linking health benefits to the kinase changes in Snell Dwarf and GHR−/− mice have been difficult to test. We therefore changed to pharmacological interventions, using drugs that have been shown to modulate mouse healthy lifespan in a sex-specific pattern.

We therefore evaluated the effects of ACA and Rapa, which extend lifespan significantly in both sexes in multiple published replicated cohorts [2, 11, 60, 61], in contrast to 17aE2, which extends lifespan only in males. We noted that ACA and Rapa downregulated mTORC1 activity in both sexes, but 17aE2 does so only in males [2], in parallel with the lifespan effects. Although the effect on mTORC1 activity was seen in both the genetic and drug models, we noted some differences; in particular, the drugs led to upregulation of 4E-BP1 protein [8], a change not seen in the two long-lived mutants [47, 62].

We hypothesized that both anti-aging drugs and anti-aging mutations might influence one or more shared pathways, “signaling hubs,” that affect transcription and/or translation within cells and tissues responsible for health maintenance, whose decline then leads to failing health, late-life diseases, and death. One of these hubs is the MAPK/ERK1/2 cascade [55]. MEK1 is the initial protein that receives signals from multiple extra-cellular receptors to activate this cascade (see Fig. 1 A). Our data on liver and kidney show an age-related upregulation of MEK1 activity (see aging effects in Fig. 2 and supplemental Table 1). This is consistent with our published data on mTORC1 [8], because mTOR signaling and MAPK/ERK1/2 share common upstream regulators including growth factors and insulin, among other receptors [27]. Downstream elements in the signaling pathway, from phosphorylation of MEK1 to eIF4E phosphorylation, are also upregulated by age (Fig. 2-5, supplemental Table 1). Rapa and ACA blunt, or possibly reverse, the age effects, leading to downregulation of this signaling cascade, starting with MEK1 activation, and followed by activation of ERK1/2, MNK and peIF4E (Figs. 2 to 5). 17aE2 treatment, however, leads to these effects only in males, in good agreement with the sexual dimorphism in mTORC1 signaling [8] and the lifespan benefits shown by these three drugs [2]. In addition, our data (Fig. 4 and Sup. Figure 2, 3) show a sexual dimorphism in the level of MNK and the phosphorylation of its downstream target eIF4E. Nothing is known as to the basis for these differences between untreated male and female mice. Nevertheless, treatments with ACA, Rapa or 17eE2 do not seem to affect any of the MAPK/ERK pathway protein levels, including MNK or eIF4E levels, in contrast to the upregulation of 4EBP1 protein [8] induced by these drugs. Recently it has been reported that reduction in MNK activity and the consequential decline in phosphorylation eIF4E improve glucose homeostasis [63], suggesting that some of the beneficial effects of these drugs and anti-aging genes may be under the control of the MAPK/ERK/eIF4E pathway. In addition, a reduction in the phosphorylation status of eIF4E (peIF4E) can upregulate the translation of a unique set of mRNAs [64, 65], suggesting that some of the effects seen in previously published data in the upregulation of CIT proteins [8] by ACA, Rapa and 17aE2 could be, in part, the result of regulation of the MAPK/ERK/eIF4E signaling by the treatments. However, a direct test of this hypothesis would require using the intervention protocols in the knockout model of the MAPK/ERK signaling, such is the MNK-KO mice [63].

We also report age-dependent increases, in liver and kidney, of the p38MAPK pathway, through which inflammatory mediators and stress signaling converge via phosphorylation and activation of MEK3 leading to a wide range of inflammatory changes and activation of stress response genes [28, 59]. This age-dependent effect is blocked by ACA, Rapa and 17aE2 in both sexes, with the effects apparent at all steps from MEK3 to MK2 (Figs. 6, 7).

We also noted effects of these anti-aging drugs on levels of specific proteins, including the long isoform of MK2, which is upregulated by ACA and Rapa in both sexes and by 17aE2 only in males. The short isoform of MK2 is downregulated by these treatments. Because translation of the MK2 isoform is dependent on the 5’UTR of the MK2 mRNA, we hypothesize that the increase in MK2-Long isoform results from upregulation of cap-independent translation by the treatments, as we have previously demonstrated for other CIT dependent targets [8].

APPs in serum are accepted surrogates as markers of inflammation and physiological stress responses and used in clinical research as indicators of acute inflammation stimulated by elevated IL-6, TNF, and related proinflammatory cytokines [66]). There is evidence that proteins from the APPs family are affected differently by age, sex, mouse strain and health status in serum samples (for example see [67, 68]). Plasma from these drug-treated mice is in very short supply, making it challenging to measure plasma levels of APPs directly, but we were able to evaluate protein and mRNA levels for three relatively abundant APPs in liver and kidney as surrogates for plasma quantitation. We found upregulation of all three APPs in older mice (consistent with effects of age on plasma levels in humans), We then measured SAP, which is produced in both liver and kidney [39] and secreted into the blood, in addition to two intracellular APP markers, HOMX2 and Cas6, whose levels could not be affected by plasma potentially present within liver and kidney samples. The liver data in Fig. 8 (Sup. Figure 5 for kidney) show that all three APPs increase with age, consistent with the age-dependent increases in p38MAPK signaling. All three of the anti-aging drugs blunt age-related increases in these three APPs markers; notably, 17aE2 does so in both males and females (see Supplemental Table 4). Parallel effects of age and drug are also seen at the transcriptional level (Fig. 8 C and Sup. Figure 5 B), implicating other downstream effectors of p38 and MK2 including perhaps Tristetraproline and ELAV-like protein 1, each of which can regulate mRNA abundance and stability [29]. The responses of females to 17aE2 suggest that the reduction of p38MAPK signaling is independent of the effects in MAPK/ERK signaling (Fig. 2-5) and mTOR signaling [8], which are more closely linked to lifespan. Our data thus suggest that these anti-aging drugs diminish p38MAPK signaling and associated proinflammatory proteins in liver and kidney. It will be useful to extend those studies to other tissues and to plasma, to test the hypothesis that Rapa, ACA and 17aE2 have similar effects on specific components of the APPs linked to proinflammatory phenotype changed by aging.

The literature suggests that other pathways, such as NFκB, can also regulate inflammation and production of APPs [69]. Thus, to test if p38MAPK can directly regulate APPs, we exposed AML-12 cells to the p38 inhibitor doramapimod or “Dor” [70]). As shown in Fig. 9A, Dor led to the expected downregulation of p38 autophosphorylation and downregulation of pMK2 signaling; in addition, it downregulated the p38-MK2 complex leading to lower levels of these proteins [43, 44]. Dor also leads to significant declines in SAP, HOMX2 and Cas6 at both protein and transcription levels (Fig. 9B). This AML-12 data corresponds well with the in vivo data (Fig. 8) and suggests that the decline in p38MAPK signaling by treatment participates in the downregulation of APP expression. Nevertheless, these results do not rule out the possible involvement of other signaling pathways in the age- and drug-dependent effects on APPs.

In this context it is interesting to note our recent report that the Snell and GHR−/− mice show, as young adults, increased numbers of anti-inflammatory (“M2”) macrophages and lower numbers of pro-inflammatory (“M1”) macrophages in both subcutaneous and visceral white adipose depots [71]. Production of cytokines generated by M1 macrophages is also diminished in these two mutants. Because aging leads to more M1 and fewer M2 cells [72] the slow-aging mutations seem to oppose this inflammatory aspect of the aging process. The possible role of kinase pathways in this shift to anti-inflammatory cells in reported fat depots is unknown [73]. Thus, it will be of high interest to see if Rapa, ACA, and 17aE2 modulate the same p38 MAPK signaling pathway in adipose tissues and other tissues and cell types, and if those also show possible sex-specificity effects.

Modulation of inflammation and stress responses often plays a central role in models of age-dependent diseases and loss of homeostasis. Several varieties of slow aging mice, including Ames, Snell Dwarf and Ghr−/− mice, have been shown to have lower levels of proinflammatory mediators [74]. It has seemed plausible that lower inflammatory tone could contribute to the exceptionally healthy lifespan of these mutant mice. Our work here shows that diminished production of inflammatory mediators is also characteristic of mice whose longevity is caused by treatment with Rapa, ACA, or 17aE2, and provides evidence for the role of age- and drug-sensitive changes in the p38-MAPK kinase cascade in this anti-inflammatory effect. The data on 17aE2 treatment in females, however, with significant downregulation of the proinflammatory p38MAPK signaling and APPs, suggest that the effects of age-related proinflammation and stress can be dissociated from effects of this drug on longevity. The pattern of sexually dimorphic effects on lifespan, in contrast, is recapitulated in the effects of these drugs on mTORC1 and MAPK/ERK signaling pathways. Furthermore, independently of the effects of these drugs on mice lifespan, our results suggest that ACA, Rapa and 17aE2 might be used as intervention methods in mice and humans where the increases in pro-inflammatory phenotypes are an important component of the declines in health and increases in morbidity.

## Conclusions

a) These three treatments are known to extend mouse lifespan and have major beneficial effects in glucose, muscle strength, metabolism and inflammation in mouse and humans. However, it is unknown how these treatments modulate intracellular pathways leading to life span extension and immunity responses to stress. The data presented here suggest that each of these agents, despite their diversity of hormonal and cell membrane targets, all converge on the regulation of two distinct MAPK pathways.

b) All three of the anti-aging drugs reduce the MEK3/p38/MK2 pathway that regulates and stimulates inflammatory responses including acute phase proteins and suggesting a downregulation of the inflammatory signaling is a key component of these beneficial effects. In addition, all three treatments also reduce the MEK1/Erk1,2/MNK/eIF4e signaling pathway, a major regulation of the translation and proteomic profiles that via specific mechanism propose in the paper.

c) In addition, because the lifespan benefit of 17α-estradiol is seen only in males, the sex-specificity of the kinase cascade leading to eIF4E phosphorylation is presented is a novel and critical mechanism in the anti-aging effects of these treatments. Furthermore, the downregulation of inflammation suggested that these treatments may have clinical value in prevention or treatment of inflammatory diseases.

## Material and methods

### Mice and diets

Genetically heterogeneous UM-HET3 mice were produced by a four-way cross between CByB6F1 mothers and C3D2F1 fathers and housed as previously described [5, 11]. Mice in breeding cages received Purina 5008 mouse chow, and weaned animals were fed Purina 5LG6. At 6 months of age, animals in different sibling groups were randomly allocated to control, ACA, Rapa or 17aE2 treatments. All diets were prepared by TestDiet, Inc., a division of Purina Mills (Richmond, IN, USA), which also produces drug/food mixtures for the NIA Interventions Testing Program . Animals in the control group remained on the 5LG6 diet. Rapamycin was given as encapsulated Rapa (L.C. Laboratories, Woburn, MA) at a dose of 14.4 mg per kilogram of 5LG6 diet (14.4 ppm) as previously described [5, 60, 75]. ACA was purchased from Spectrum Chemical Mfg. Corp. (Gardena, CA) and used at a concentration of 1000 mg of ACA/kg of diet (1000 ppm) as previously described [76]. 17aE2 was purchased from Steraloids Inc. (Newport, RI, USA) and used at a dose of 14.4 mg per kilogram of 5LG6 diet (14.4 ppm) as previously described [76]. All these methods followed those used by the NIA Interventions Testing Program.

### Tissue harvest, Western blots, and qRT-PCR analysis

Tissues were harvested at the age indicated in Supplemental Fig. 1; tissues were harvested during the morning after 18 h of fasting. Tissues were flash frozen with liquid nitrogen and stored at − 80 °C. Cell lysates were prepared, after which equal amounts of protein were loaded for Western blot analysis of actin. Then samples from each mouse were normalized to the same actin concentration present in young female untreated controls and the specific proteins or phospho-sites measured by Western blots as described previously [52]. Each Western Blot contained at least one sample for each group (young, old, ACA, Rapa or 17aE2 treatments for males and females). Data on band intensity was normalized with respect to young female controls for each blot, to allow combination of results from multiple Western blots. The specific antibodies used in the analysis are listed in Supplemental Table 5. qRT-PCR from the same set of samples was performed as previously described [52]. qRT-PCR probe sequences are described in Supplemental Table 6.

### Statistics

All statistics were carried out in GraphPad Prism version 7.0. Data from animals treated with ACA, Rapa and 17aE2 were analyzed separately, but the same young and aged control animals were used in each analysis. To normalize between different western blots, we use young females as a reference as we previously described [52]. After normalization the full data for each measured parameter was analyzed by two-factor ANOVA, using a general linear model and a full factorial model, which included an effect of treatment (comparing control to either ACA or Rapa), an effect of sex (male or female), and an interaction between sex and treatment. Because for ACA and Rapa we found no significant interaction between treatments and sex, we pooled the data of male and females set for each group to perform statistical analysis. For 17aE2, however, most endpoints had a significant Sex x Drug interaction term, and therefore males and females were analyzed separately. In the same way, the absence of a (Sex x Age) interaction term for comparisons of young and aged controls allowed us to pool across sex for tests of age effects on each endpoint. Each analysis of drug or sex effect then began with a one-way ANOVA followed by t-tests to evaluate specific contrasts of interest, i.e., Young vs Old controls, and Old controls vs each drug-treated group. Significance was evaluated using a criterion of *p* = 0.05 without adjustment for multiple comparisons.

## Supplementary Information

**Additional file 1.** Supplemental Figures Immunity Aging Garcia et al.

**Additional file 2: Supplemental Table 1.** Statistical analysis of age and treatments effects in the ERK signaling pathway. **Supplemental Table 2.** Statistical analysis of age and treatments effects in the p38 MAPK signaling pathway. **Supplemental Table 3.** Statistical analysis of age and treatments effects in the Levels of Acute Phase Proteins. **Supplemental Table 4.** Statistical analysis of age and treatments effects in the Levels of Acute Phase Proteins mRNAs. **Supplemental Table 5.** Source of Antibodies. **Supplemental Table 6.** qRT-PCR.

## Data Availability

All original material and data is the results of this study would be available upon request to the authors, while original immunoblots can be found in the following drop box: https://www.dropbox.com/sh/285n4mc1zjy5dw1/AAAT-lam7K4kdNDVmCjaTBpZa?dl=0

## Abbreviations

ACA: Acarbose
Rapa: Rapamycin
17aE2: 17-α Estradiol
MEK1: Dual specificity mitogen-activated protein kinase kinase 1
ERK1: Mitogen-activated protein kinase 3
ERK2: Mitogen-activated protein kinase 1
MNK1: MAP kinase-interacting serine/threonine-protein kinase 1
MNK2: MAP kinase-interacting serine/threonine-protein kinase 2
eIF4E: Eukaryotic translation initiation factor 4E
MEK3: Dual specificity mitogen-activated protein kinase kinase 3
p38: Mitogen-activated protein kinase 11 and Mitogen-activated protein kinase 14
MK2: MAP kinase-activated protein kinase 2
SAP: Serum amyloid P-component
HMOX1: Heme oxygenase 1
HMOX2: Heme oxygenase 2
Cas6: Caspase 6
Dor: Doramapimod
