# Supplementary material for: Rapamycin, Acarbose and 17α-estradiol share common mechanisms regulating the MAPK pathways involved in intracellular signaling and inflammation
Source: Immun Ageing. 2022 Feb 1;19:8. doi: 10.1186/s12979-022-00264-1 (PMC8805398; doi:10.1186/s12979-022-00264-1)
Supplement: Supplementary file 1 — Additional file 1. Supplemental Figures Immunity Aging Garcia et al. [file 12979_2022_264_MOESM1_ESM.pdf]

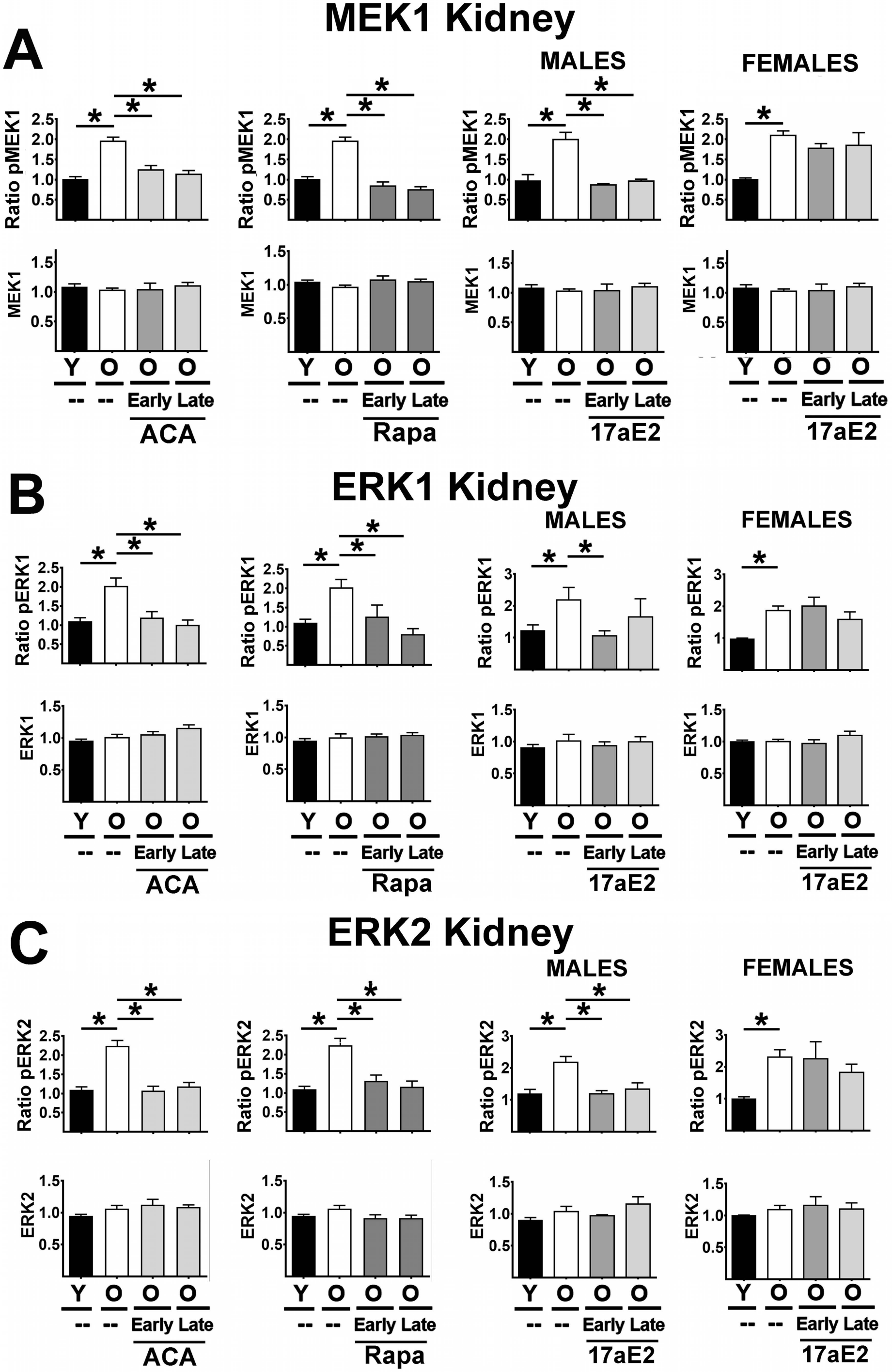

Supplemental Figure 1. Bar graphs represent the mean ± SEM of MEK1 and ERK1/2 protein levels and phosphorylation status in kidney samples as described in Fig. 2

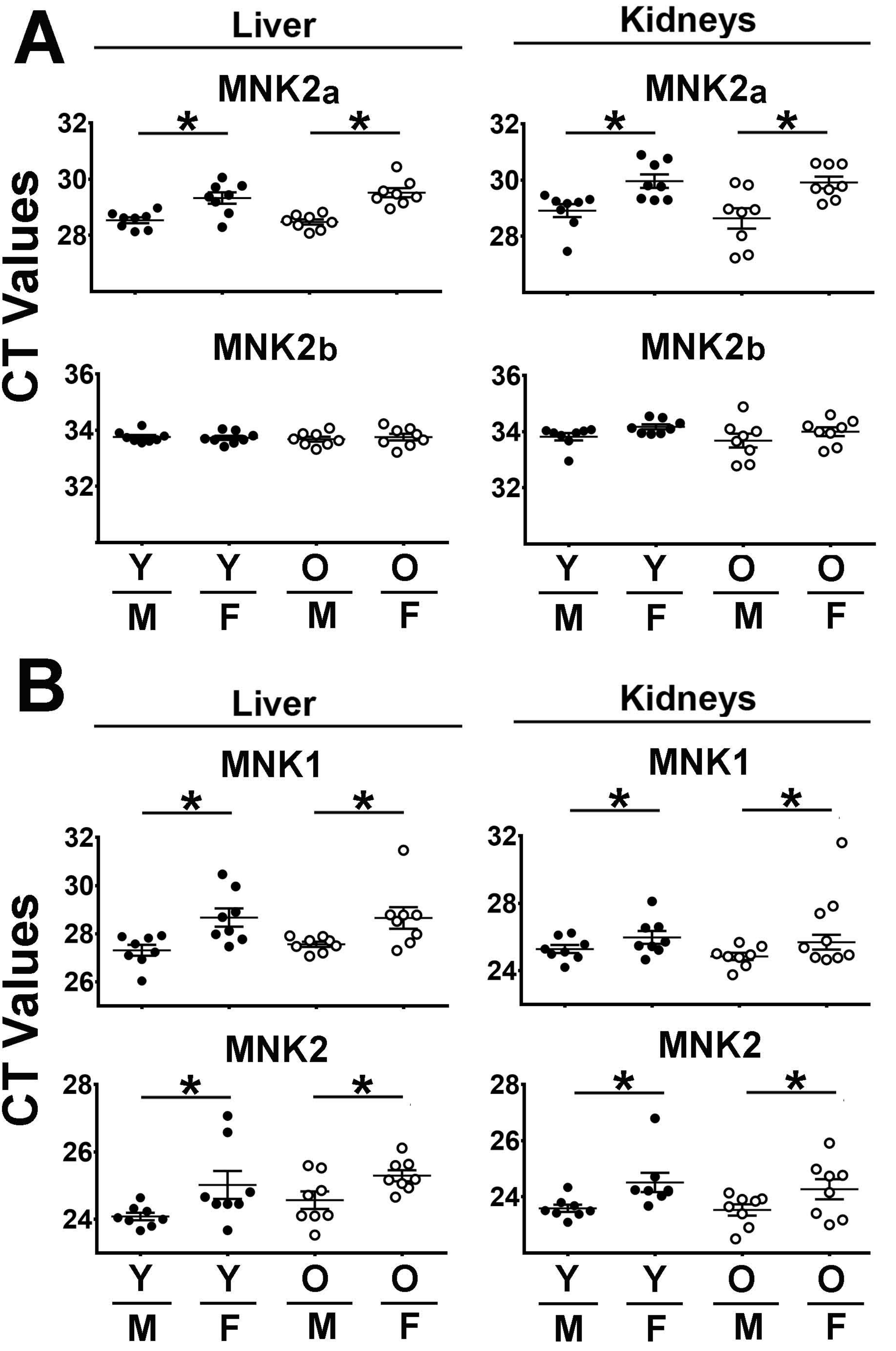

Supplemental Figure 2. A) Effect of age in the mRNA levels of the MNK2 isoforms in liver and kidney samples as described in Fig. 3 B) Sex and age effects in mRNA of MNK1 and MNK2 levels as described in Fig 3

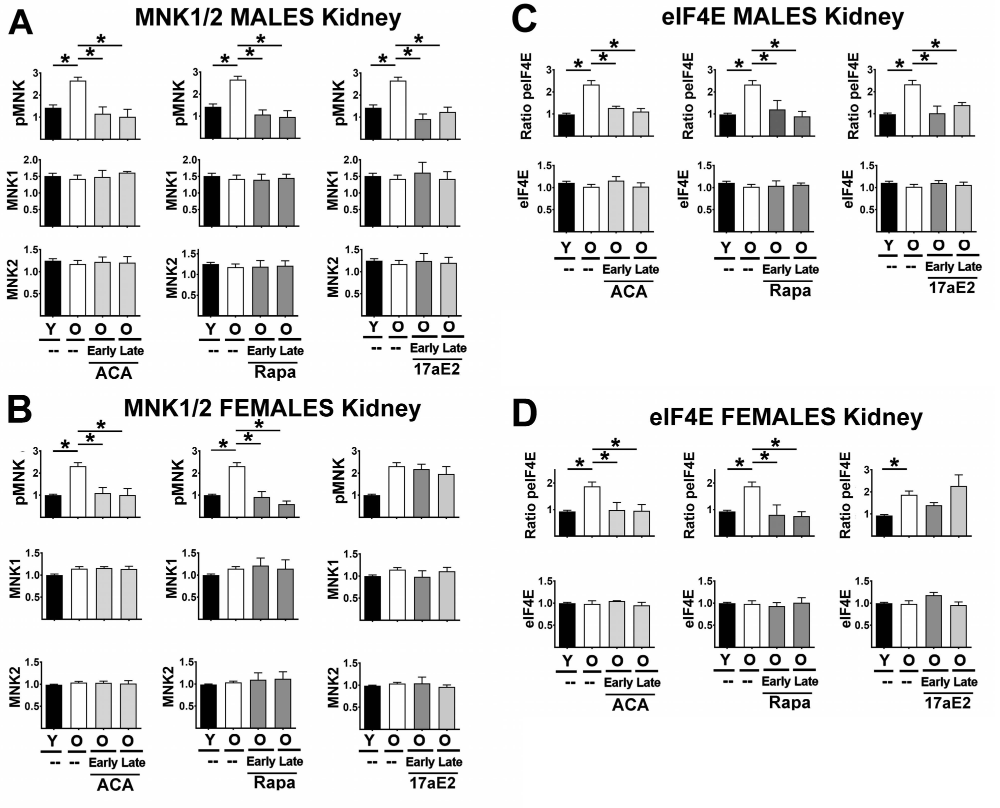

Supplemental Figure 3. Bar graphs represent mean SEM for pMNK and peIF4E plus respective protein levels in kidney as described in Figure 3 and 4

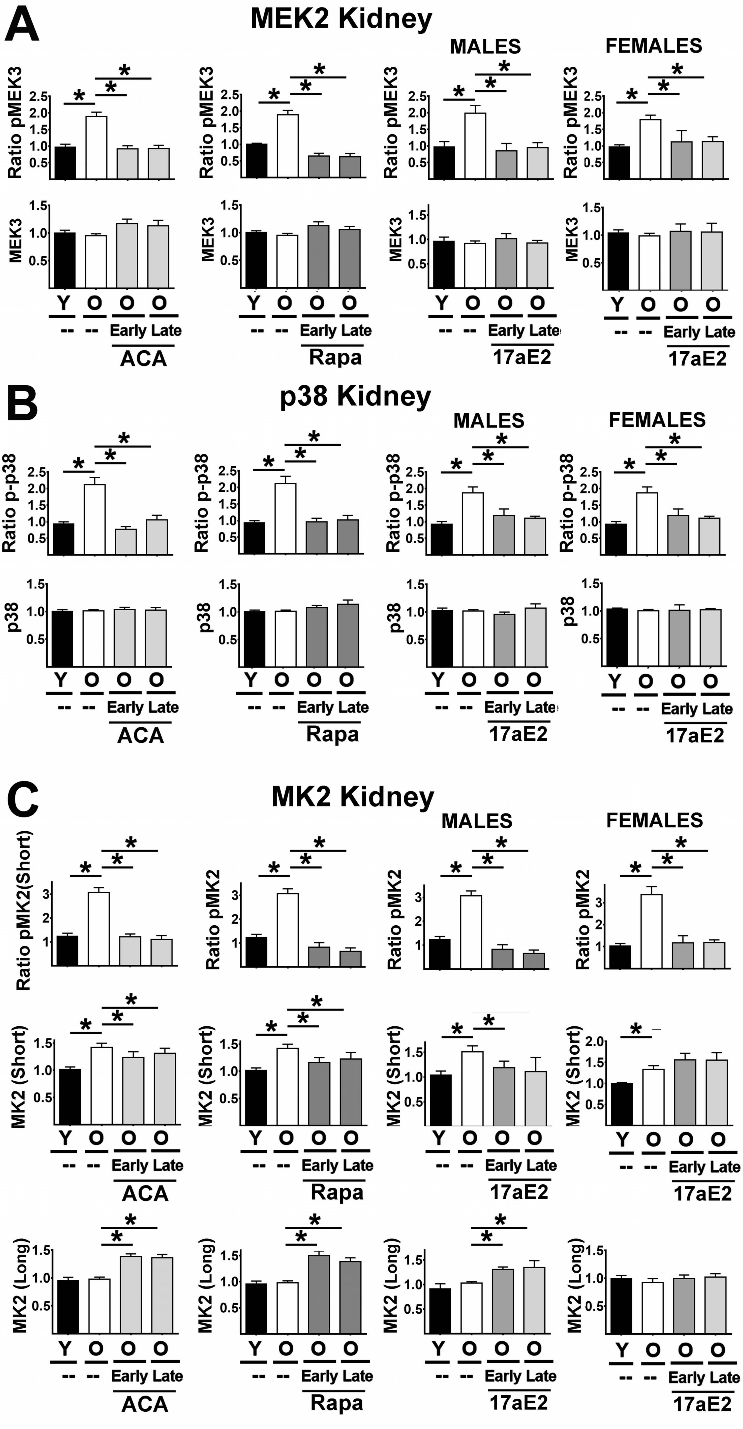

Supplemental Figure 4. Bar graphs represent the mean SEM for ratios of pMEK3, p38 and pMK2 plus respective protein levels in kidney as described in Fig 5 and 6.

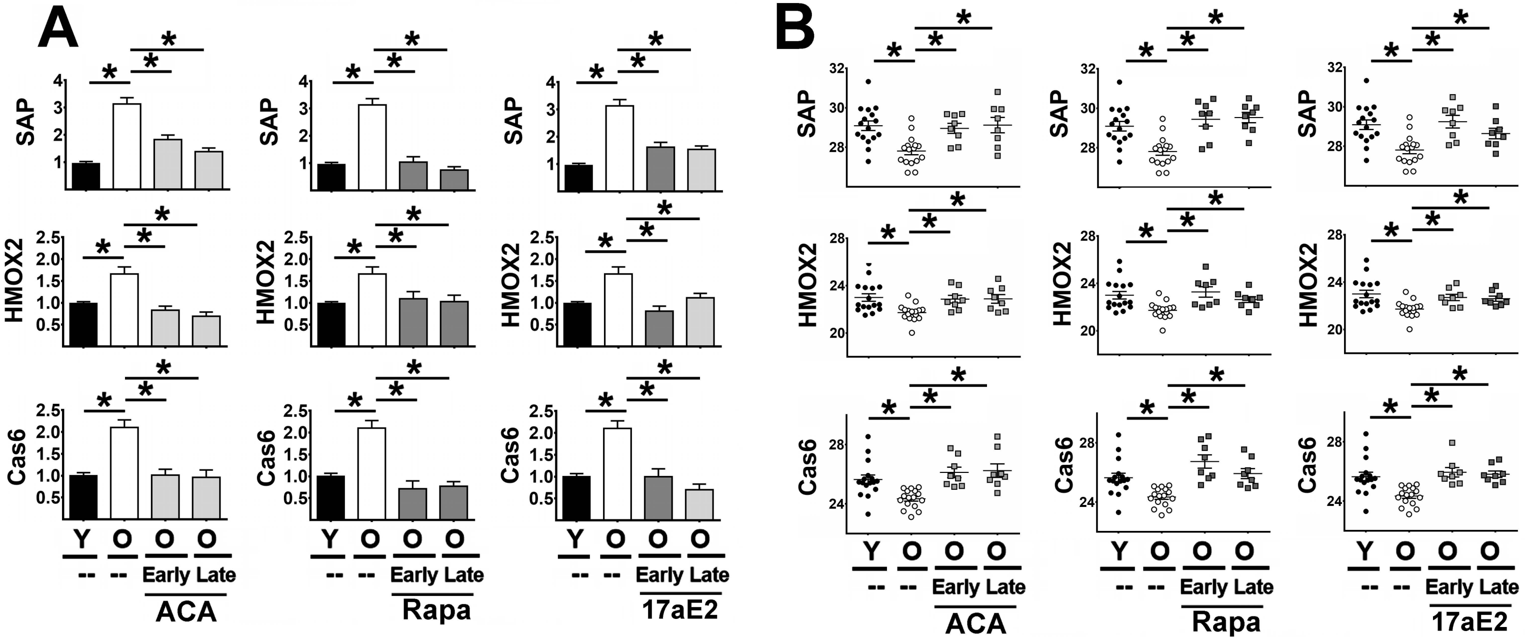

Supplemental Figure 5. Acute Phase Proteins levels in Kidneys as described in Figure 7
