# Supplementary material for: Rapamycin, Acarbose and 17α-estradiol share common mechanisms regulating the MAPK pathways involved in intracellular signaling and inflammation
Source: Immun Ageing. 2022 Feb 1;19:8. doi: 10.1186/s12979-022-00264-1 (PMC8805398; doi:10.1186/s12979-022-00264-1)
Supplement: Supplementary file 2 — Additional file 2: Supplemental Table 1. Statistical analysis of age and treatments effects in the ERK signaling pathway. Supplemental Table 2. Statistical analysis of age and treatments effects in the p38 MAPK signaling pathway. Supplemental Table 3. Statistical analysis of age and treatments effects in the Levels of Acute Phase Proteins. Supplemental Table 4. Statistical analysis of age and treatments effects in the Levels of Acute Phase Proteins mRNAs. Supplemental Table 5. Source of Antibodies. Supplemental Table 6. qRT-PCR. [file 12979_2022_264_MOESM2_ESM.pdf]

Supplemental Table 1. Statistical analysis of age and treatments effects in the ERK signaling pathway.

					ACA				Rapa				17aE2				17aE2 by Sex				
Protein	Tissue	Analysis	Significant AGE Effect	Sex Interaction	Significance Early ACA	Sex Interaction ACA Early	Significance Late ACA	Sex Interaction ACA Late	Significance Early Rapa	Sex Interaction Rapa Early	Significanc e Late Rapa	Sex Interaction Rapa Late	Significance Early 17αE2	Sex Interaction Early 17aE2	Significance Late 17aE2	Sex Interaction Late 17aE2	Analysis	Males Early 17aE2	Males Late 17aE2	Females Early 17aE2	Females Late 17aE2
Ratio P-MEK1	Liver	Two-Way ANOVA	YES Increases (p<0.0001)	NO	YES Declines (p<0.0001)	NO	YES Declines (p<0.0001)	NO	YES Declines (p<0.0001)	NO	YES Declines (p<0.0001)	NO	Analysis Separate by sex	YES	Analysis Separate by sex	YES	One-Way ANOVA	Yes Declines (p=0.0022)	Yes Declines (p=0.0001)	NO	NO
Ratio P-MEK1	Kidney	Two-Way ANOVA	YES Increases (p<0.0001)	NO	YES Declines (p<0.0001)	NO	YES Declines (p<0.0001)	NO	YES Declines (p<0.0001)	NO	YES Declines (p<0.0001)	NO	Analysis Separate by sex	YES	Analysis Separate by sex	YES	One-Way ANOVA	YES Declines (p=0.0005)	YES Declines (p=0.0010)	NO	NO
MEK1	Liver	Two-Way ANOVA	NO	NO	NO	NO	NO	NO	NO	NO	NO	NO	NO	NO	NO	NO	NO	NO	NO	NO	NO
MEK1	Kidney	Two-Way ANOVA	NO	NO	NO	NO	NO	NO	NO	NO	NO	NO	NO	NO	NO	NO	NO	NO	NO	NO	NO
Ratio P-ERK1	Liver	Two-Way ANOVA	YES Increases (p<0.0001)	NO	YES Declines (p=0.0026)	NO	YES Declines (p=0.0005)	NO	YES Declines (p=0.0025)	NO	YES Declines (p=0.0017)	NO	Analysis Separate by sex	YES	Analysis Separate by sex	NO	One-Way ANOVA	Yes Declines (p=0.0129)	Yes Declines (p=0.018)	NO	NO
Ratio P-ERK1	Kidney	Two-Way ANOVA	YES Increases (p=0.0005)	NO	YES Declines (p=0.0156)	NO	YES Declines (p=0.0001)	NO	YES Declines (p=0.0480)	NO	YES Declines (p=0.0009)	NO	Analysis Separate by sex	YES	Analysis Separate by sex	NO	One-Way ANOVA	Yes Declines (p=0.05)	NO Declines (p=0.06)	NO	NO
ERK1	Liver	Two-Way ANOVA	NO	NO	NO	NO	NO	NO	NO	NO	NO	NO	NO	NO	NO	NO	NO	NO	NO	NO	NO
ERK1	Kidney	Two-Way ANOVA	NO	NO	NO	NO	NO	NO	NO	NO	NO	NO	NO	NO	NO	NO	NO	NO	NO	NO	NO
Ratio P-ERK2	Liver	Two-Way ANOVA	YES Increases (p<0.0001)	NO	YES Declines (p=0.0021)	NO	YES Declines (p<0.0001)	NO	YES Declines (p=0.0004)	NO	YES Declines (p=0.0004)	NO	Analysis Separate by sex	YES	Analysis Separate by sex	NO	One-Way ANOVA	Yes Declines (p=0.022)	Yes Declines (p=0.0354)	NO	NO
Ratio P-ERK2	Kidney	Two-Way ANOVA	YES Increases (p=0.0122)	NO	YES Declines (p<0.0001)	NO	YES Declines (p<0.0001)	NO	YES Declines (p=0.0009)	NO	YES Declines (p=0.0002)	NO	Analysis Separate by sex	YES	Analysis Separate by sex	NO	One-Way ANOVA	YES Declines (p=0.0051)	YES Declines (p=0.0145)	NO	NO
ERK2	Liver	Two-Way ANOVA	NO	NO	NO	NO	NO	NO	NO	NO	NO	NO	NO	NO	NO	NO	NO	NO	NO	NO	ND
ERK2	Kidney	Two-Way ANOVA	NO	NO	NO	NO	NO	NO	NO	NO	NO	NO	NO	NO	NO	NO	NO	NO	NO	NO	NO
Ratio P-MNK	Liver	Two-Way ANOVA	YES Increases (p<0.0001)	NO	YES Declines (p<0.0001)	NO	YES Declines (p<0.0001)	NO	YES Declines (p<0.0001)	NO	YES Declines (p<0.0001)	NO	Analysis Separate by sex	YES	Analysis Separate by sex	YES	One-Way ANOVA	Yes Declines (p=0.0008)	Yes Declines (p=0.0002)	Yes Declines (p=0.0453)	NO
Ratio P-MNK	Kidney	Two-Way ANOVA	YES Increases (p<0.0001)	NO	YES Declines (p<0.0001)	NO	YES Declines (p<0.0001)	NO	YES Declines (p<0.0001)	NO	YES Declines (p<0.0001)	NO	Analysis Separate by sex	YES	Analysis Separate by sex	YES	One-Way ANOVA	YES Declines (p<0.0001)	YES Declines (p=0.0003)	NO	NO
MNK1	Liver	Two-Way ANOVA	NO	NO	NO	NO	NO	NO	NO	NO	NO	NO	NO	NO	NO	NO	NO	NO	NO	NO	ND
MNK1	Kidney	Two-Way ANOVA	NO	NO	NO	NO	NO	NO	NO	NO	NO	NO	NO	NO	NO	NO	NO	NO	NO	NO	NO
MNK2	Liver	Two-Way ANOVA	NO	NO	NO	NO	NO	NO	NO	NO	NO	NO	NO	NO	NO	NO	NO	NO	NO	NO	ND
MNK2	Kidney	Two-Way ANOVA	NO	NO	NO	NO	NO	NO	NO	NO	NO	NO	NO	NO	NO	NO	NO	NO	NO	NO	NO
Ratio P-eIF4E	Liver	Two-Way ANOVA	YES Increases (p<0.0001)	NO	YES Declines (p=0.0047)	NO	YES Declines (p=0.0023)	NO	YES Declines (p=0.0013)	NO	YES Declines (p<0.0001)	NO	Analysis Separate by sex	NO	Analysis Separate by sex	YES	One-Way ANOVA	Yes Declines (p=0.0172)	Yes Declines (p=0.0068)	Yes Declines (p=0.0421)	NO
Ratio P-eIF4E	Kidney	Two-Way ANOVA	YES Increases (p<0.0001)	NO	YES Declines (p=0.0001)	NO	YES Declines (p<0.0001)	NO	YES Declines (p=0.0003)	NO	YES Declines (p<0.0001)	NO	Analysis Separate by sex	NO	Analysis Separate by sex	YES	One-Way ANOVA	YES Declines (p=0.0031)	YES Declines (p=0.0008)	NO	NO
eIF4E	Liver	Two-Way ANOVA	NO	NO	NO	NO	NO	NO	NO	NO	NO	NO	NO	NO	NO	NO	NO	NO	NO	NO	ND
eIF4E	Kidney	Two-Way ANOVA	NO	NO	NO	NO	NO	NO	NO	NO	NO	NO	NO	NO	NO	NO	NO	NO	NO	NO	NO

Supplemental Table 2. Statistical analysis of age and treatments effects in the p38 MAPK signaling pathway.

					ACA				Rapa				17aE2			
Protein	Tissue	Analysis	Significant AGE Effect	Sex Interaction	Significance Early ACA	Sex Interaction ACA Early	Significance Late ACA	Sex Interaction ACA Late	Significance Early Rapa	Sex Interaction Rapa Early	Significanc e Late Rapa	Sex Interaction Rapa Late	Significance Early 17aE2	Sex Interaction Early 17aE2	Significance Late 17aE2	Sex Interaction Late 17aE2
Ratio P-MEK3	Liver	Two-Way ANOVA	YES Increases (p=0.0004)	NO	YES Declines (p=0.0466)	NO	YES Declines (p=0.0003)	NO	YES Declines (p=0.0021)	NO	YES Declines (p=0.0007)	NO	YES Declines (p=0.0433)	NO	YES Declines (p=0.0025)	NO
Ratio P-MEK3	Kidney	Two-Way ANOVA	YES Increases (p<0.0001)	NO	YES Declines (p=0.0002)	NO	YES Declines (p<0.0001)	NO	YES Declines (p<0.0001)	NO	YES Declines (p<0.0001)	NO	YES Declines (p=0.0013)	NO	YES Declines (p=0.0006)	NO
MEK3	Liver	Two-Way ANOVA	NO	NO	NO	NO	NO	NO	NO	NO	NO	NO	NO	NO	NO	NO
MEK3	Kidney	Two-Way ANOVA	NO	NO	NO	NO	NO	NO	NO	NO	NO	NO	NO	NO	NO	NO
Ratio P-p38	Liver	Two-Way ANOVA	YES Increases (p=0.0002)	NO	YES Declines (p=0.0003)	NO	YES Declines (p=0.0157)	NO	YES Declines (p=0.0002)	NO	YES Declines (p=0.015)	NO	YES Declines (p=0.0038)	NO	YES Declines (p=0.0212)	NO
Ratio P-p38	Kidney	Two-Way ANOVA	YES Increases (p<0.0001)	NO	YES Declines (p<0.0001)	NO	YES Declines (p<0.0001)	NO	YES Declines (p<0.0001)	NO	YES Declines (p=0.0002)	NO	YES Declines (p=0.0008)	NO	YES Declines (p=0.0015)	NO
p38	Liver	Two-Way ANOVA	NO	NO	NO	NO	NO	NO	NO	NO	NO	NO	NO	NO	NO	NO
p38	Kidney	Two-Way ANOVA	NO	NO	NO	NO	NO	NO	NO	NO	NO	NO	NO	NO	NO	NO
Ratio P-MK2	Liver	Two-Way ANOVA	YES Increases (p<0.0001)	NO	YES Declines (p<0.0001)	NO	YES Declines (p=0.0005)	NO	YES Declines (p<0.0001)	NO	YES Declines (p<0.0001)	NO	YES Declines (p=0.0001)	NO	YES Declines (p=0.0002)	NO
Ratio P-MK2	Kidney	Two-Way ANOVA	YES Increases (p<0.0001)	NO	YES Declines (p=0.0001)	NO	YES Declines (p<0.0001)	NO	YES Declines (p<0.0001)	NO	YES Declines (p<0.0001)	NO	YES Declines (p<0.0001)	NO	YES Declines (p<0.0001)	NO
MK2 (Long)	Liver	Two-Way ANOVA	NO	NO	YES Increases (p<0.0001)	NO	YES Increases (p,0.0001)	NO	YES Increases (p<0.0001)	NO	YES Increases (p<0.0001)	NO	YES Increases (p=0.0074)	YES	YES Increases (p=0.0008)	YES
MK2 (Long)	Kidney	Two-Way ANOVA	NO	NO	YES Increases (p<<0.0001)	NO	YES Increases (p<<0.0001)	NO	YES Increases (p<<0.0001)	NO	YES Increases (p<<0.0001)	NO	YES Increases (p=0.0092)	YES	YES Increases (p=0.0118)	YES
MK2 (Short)	Liver	Two-Way ANOVA	YES Increases (p=0.0047)	NO	YES Declines (p=0.0002)	NO	YES Declines (p<0.0001)	NO	YES Declines (p<0.0001)	NO	YES Declines (p<0.0001)	NO	YES Declines (p=0.003)	YES	YES Declines (p=0.0097)	YES
MK2 (Short)	Kidney	Two-Way ANOVA	YES Increases (p<0.0001)	NO	YES Declines (p=0.05)	NO	YES Declines (p=0.002)	NO	YES Declines (p=0.05)	NO	YES Declines (p=0.05)	NO	NO	YES	NO	YES

Supplemental Table 3. Statistical analysis of age and treatments effects in the Levels of Acute Phase Proteins.

					ACA				Rapa				17aE2			
Protein	Tissue	Analysis	Significant AGE Effect	Sex Interaction	Significance Early ACA	Sex Interaction ACA Early	Significance Late ACA	Sex Interaction ACA Late	Significance Early Rapa	Sex Interaction Rapa Early	Significanc e Late Rapa	Sex Interaction Rapa Late	Significance Early 17aE2	Sex Interaction Early 17aE2	Significance Late 17aE2	Sex Interaction Late 17aE2
SAP	Liver	Two-Way ANOVA	YES Increases (p<0.0001)	NO	YES Declines (p=0.0028)	NO	YES Declines (p=0.0018)	NO	YES Declines (p=0.0035)	NO	YES Declines (p=0.0018)	NO	YES Declines (p=0.0018)	NO	YES Declines (p=0.0038)	NO
SAP	Kidney	Two-Way ANOVA	YES Increases (p<0.0001)	NO	YES Declines (p=0.0014)	NO	YES Declines (p<0.0001)	NO	YES Declines (p<0.0001)	NO	YES Declines (p<0.0001)	NO	YES Declines (p=0.0003)	NO	YES Declines (p=0.0002)	NO
HMOX2	Liver	Two-Way ANOVA	YES Increases (p<0.0001)	NO	YES Declines (p=0.0023)	NO	YES Declines (p=0.0017)	NO	YES Declines (p<0.0001)	NO	YES Declines (p<0.0001)	NO	YES Declines (p=0.0017)	NO	YES Declines (p=0.0004)	NO
HMOX2	Kidney	Two-Way ANOVA	YES Increases (p=0.0005)	NO	YES Declines (p=0.0033)	NO	YES Declines (p<0.0001)	NO	YES Declines (p=0.0465)	NO	YES Declines (p=0.0257)	NO	YES Declines (p=0.0013)	NO	YES Declines (p=0.0006)	NO
Caspase 6	Liver	Two-Way ANOVA	YES Increases (p<0.0001)	NO	YES Declines (p=0.0003)	NO	YES Declines (p<0.0001)	NO	YES Declines (p<0.0001)	NO	YES Declines (p<0.0001)	NO	YES Declines (p<0.0001)	NO	YES Declines (p<0.0001)	NO
Caspase 6	Kidney	Two-Way ANOVA	YES Increases (p<0.0001)	NO	YES Declines (p=0.001)	NO	YES Declines (p<0.0001)	NO	YES Declines (p=0.0001)	NO	YES Declines (p=0.0001)	NO	YES Declines (p=0.001)	NO	YES Declines (p<0.0001)	NO

Supplemental Table 4. Statistical analysis of age and treatments effects in the Levels of Acute Phase Proteins mRNAs.

mRNA	Tissue	Analysis	Significant AGE Effect in Ct Values	Sex Interaction	Significance Early ACA Effects in Ct Values	Sex Interaction ACA Early	Significance Late ACA Effects in Ct Values	Sex Interaction ACA Late	Significance Early Rapa Effects in Ct Values	Sex Interaction ACA Early	Significanc e Late Rapa Effects in Ct Values	Sex Interaction ACA Late	Significance Early 17aE2 Effects in Ct Values	Sex Interaction ACA Early	Significance Late 17aE2 Effects in Ct Values	Sex Interaction ACA Late
SAP	Liver	Two-Way ANOVA	YES Declines ($p<0.0001$)	NO	YES Increases ($p=0.001$)	NO	YES Increases ($p=0.0003$)	NO	YES Increases ($p=0.0368$)	NO	YES Increases ($p=0.0030$)	NO	YES Increases ($p=0.0002$)	NO	YES Increases ($p=0.0048$)	NO
SAP	Kidney	Two-Way ANOVA	YES Declines ($p=0.0002$)	NO	YES Declines ($p=0.001$)	NO	YES Declines ($p=0.0021$)	NO	YES Declines ($p=0.0001$)	NO	YES Declines ($p<0.0001$)	NO	YES Declines ($p=0.0002$)	NO	YES Declines ($p=0.0178$)	NO
HMOX2	Liver	Two-Way ANOVA	YES Declines ($p<0.0001$)	NO	YES Increases ($p=0.0002$)	NO	YES Increases ($p<0.0001$)	NO	YES Increases ($p=0.0006$)	NO	YES Increases ($p<0.0001$)	NO	YES Increases ($p<0.0001$)	NO	YES Increases ($p=0.0001$)	NO
HMOX2	Kidney	Two-Way ANOVA	YES Declines ($p=0.0015$)	NO	YES Declines ($p=0.0026$)	NO	YES Declines ($p=0.0028$)	NO	YES Declines ($p=0.0008$)	NO	YES Declines ($p=0.006$)	NO	YES Declines ($p=0.0013$)	NO	YES Declines ($p=0.0058$)	NO
Caspase 6	Liver	Two-Way ANOVA	YES Declines ($p<0.0001$)	NO	YES Increases ($p=0.0006$)	NO	YES Increases ($p=0.0024$)	NO	YES Increases ($p=0.0002$)	NO	YES Increases ($p<0.0001$)	NO	YES Increases ($p=0.0011$)	NO	YES Increases ($p=0.0030$)	NO
Caspase 6	Kidney	Two-Way ANOVA	YES Declines ($p=0.0008$)	NO	YES Declines ($p<0.0001$)	NO	YES Declines ($p<0.0001$)	NO	YES Declines ($p<0.0001$)	NO	YES Declines ($p=0.0001$)	NO	YES Declines ($p<0.0001$)	NO	YES Declines ($p<0.0001$)	NO

Supplemental Table 5. Source of Antibodies

Antibody	Company	Cat ID:
MEK1	Cell Signaling	#12671
Phospho-MEK1 (Ser217)	Cell Signaling	#9154
ERK1/2	Cell Signaling	#4695
Phospho-ERK1/2 (Thr202)	Cell Signaling	#9101
MNK1	Cell Signaling	#2195
MNK2	Bioss	#17697R
Phospho-MNK (Thr197)	Invitrogen	#700242
eIF4E	Protein Tech	#11332
Phospho-eIF4E (Ser209)	Boster	P00135
MEK3	Cell Signaling	#7421
Phospho-MEK3 (Ser189)	Cell Signaling	#12280
p38 MAPK Total	Cell Signaling	#8690
Phospho-p38 (Th180/192)	Cell Signaling	#9211
MK2	Cell Signaling	#12155
Phospho-MK2 (Th334)	Cell Signaling	#3007
Caspase-6	Invitrogen	#32201
Serum amyloid P	R&D systems	Af2558
HMOX2	Protein Tech	#14817-1-AP

Supplemental Table 6. qRT-PCR

Probe		Mouse Sequence probes
**Cas6**	Forward	CTCGCAGGTTTTCAGATCTAGG
	Reverse	GGCTCAGGAAGACACAGATG
**SAP**	Forward	GAATTGGCAGGCTCTTAACTATG
	Reverse	CCTTGACCTCTTACACATCGG
**HMOX2**	Forward	TCAGCCACAATGTCTTCAGAG
	Reverse	TCGGTCATGTGCTTCCTTG
**p38 MAPK alfa**	Forward	GTGATTGGTCTGTTGGATGTG
	Reverse	TGAGAAACTGAACGTGGTCG
**p38 MAPK beta**	Forward	TGTCTCGCCCTTTCCAATC
	Reverse	AGGTACACTTCGCTGAAATCC
**MNK2**	Forward	GGGAGGTGGAGATGCTGTA
	Reverse	CTATGGATGTGGCTTAGGATGG
**MNK2a**	Forward	GCTGCGACCTGTGGAGCCTGGG
	Reverse	GATGGGAGGGTCAGGCGTGGTC
**MNK2b**	Forward	GCTGCGACCTGTGGAGCCTGGG
	Reverse	GAGGAGGAAGTGACTGTCCCAC
**MNK1**	Forward	TGTGACTTTGACTTGGGCAG
	Reverse	GTCATAGAAAGTAGCCTCGTCC
**MK2**	Forward	CCCCTGGATCATGCAATCTAC
	Reverse	TCTGCTCATAGTCAACACGC
